# Design of a FAIR digital data health infrastructure in Africa for COVID‐19 reporting and research

**DOI:** 10.1002/ggn2.10050

**Published:** 2021-06-11

**Authors:** Mirjam van Reisen, Francisca Oladipo, Mia Stokmans, Mouhamed Mpezamihgo, Sakinat Folorunso, Erik Schultes, Mariam Basajja, Aliya Aktau, Samson Yohannes Amare, Getu Tadele Taye, Putu Hadi Purnama Jati, Kudakwashe Chindoza, Morgane Wirtz, Meriem Ghardallou, Gertjan van Stam, Wondimu Ayele, Reginald Nalugala, Ibrahim Abdullahi, Obinna Osigwe, John Graybeal, Araya Abrha Medhanyie, Abdullahi Abubakar Kawu, Fenghong Liu, Katy Wolstencroft, Erik Flikkenschild, Yi Lin, Joëlle Stocker, Mark A. Musen

**Affiliations:** ^1^ Leiden University Leiden Netherlands; ^2^ Leiden University Medical Centre (LUMC) Leiden University Leiden Netherlands; ^3^ Leiden Institute of Advanced Computer Science (LIACS) Leiden University Leiden Netherlands; ^4^ Faculty of Humanities and Digital Sciences Tilburg University Tilburg Netherlands; ^5^ Kampala International University Kampala Uganda; ^6^ Department of Computer Science Olabisi Onabanjo University Ago Iwoye Nigeria; ^7^ Go‐FAIR Foundation Leiden Netherlands; ^8^ School of Public Health Mekelle University Mek'ele Ethiopia; ^9^ Department of Health informatics, School of Public Health Mekelle University Mek'ele Ethiopia; ^10^ Badan Pusat Statistik Central Jakarta Indonesia; ^11^ Department of Computer Science Great Zimbabwe University Masvingo Zimbabwe; ^12^ Department of Community Medicine Université de Sousse Sousse Tunisia; ^13^ SolidarMed Masvingo Zimbabwe; ^14^ Department of Biostatistics and Epidemiology, School of Public health College of Health Sciences Addis Ababa University Addis Ababa Ethiopia; ^15^ Tangaza University College Nairobi Kenya; ^16^ Ibrahim Badamasi Babangida University Lapai Nigeria; ^17^ Stanford Center for Biomedical Informatics Research Stanford University Stanford California USA; ^18^ Department of Reproductive health, School of Public Health Mekelle University Mek'ele Ethiopia; ^19^ Chinese Academy of Science Beijing China; ^20^ Department of Geosciences Utrecht University Utrecht Netherlands

**Keywords:** data science, ethical, health information, knowledge capture, legal and social implications, medical informatics, population genetics, social health care, viral genomes

## Abstract

The limited volume of COVID‐19 data from Africa raises concerns for global genome research, which requires a diversity of genotypes for accurate disease prediction, including on the provenance of the new SARS‐CoV‐2 mutations. The Virus Outbreak Data Network (VODAN)‐Africa studied the possibility of increasing the production of clinical data, finding concerns about data ownership, and the limited use of health data for quality treatment at point of care. To address this, VODAN Africa developed an architecture to record clinical health data and research data collected on the incidence of COVID‐19, producing these as human‐ and machine‐readable data objects in a distributed architecture of locally governed, linked, human‐ and machine‐readable data. This architecture supports analytics at the point of care and—through data visiting, across facilities—for generic analytics. An algorithm was run across FAIR Data Points to visit the distributed data and produce aggregate findings. The FAIR data architecture is deployed in Uganda, Ethiopia, Liberia, Nigeria, Kenya, Somalia, Tanzania, Zimbabwe, and Tunisia.

## Graphical abstract

Organization of clinical health data interoperability with research communities. Interoperability of clinical health data in residence facilitating data visualization at point of care and investigation of data across facilities through data visiting.

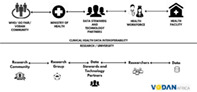

## 1 INTRODUCTION

The trend in data management is to invest in computational systems, because researchers and practitioners increasingly rely on support to deal with the enormous volume of data. Such systems, usually based on cloud computing, often ignore the ecology of data, assuming that data is generalizable. On the other hand, there is concern about the diminishing diversity of data when separated from its data subjects and data ecology^1^ related to validity,^2^ which is especially relevant for organisms,^3^ including humans, who by nature function as part of social processes. Bronfenbrenner^4^ ^(p. 513)^ found that “not only the immediate settings containing the developing person but also the larger social contexts, both formal and informal, in which these settings are embedded” are relevant as the context for interpreting data, which is recognized in cross‐cultural approaches.^5^ Understanding data in contextual settings requires a cumulative design approach^6^ that recognizes that data are always situated in time and space and the result of social processes.

The problem of data diversity in computational data management systems starts with the differences in the ecologies from which the data is collected. As a result of social processes, there may be an absence or incompleteness of data due to the exclusion of marginalized groups or geographies from the data, which has the effect of slowing down progress in areas such as the study of human genetics.^7^ While the inclusion of data from non‐Western settings may be important, involving the African continent, for instance, can be complex.^8^ Despite the complexity involved in developing computational data management systems that are inclusive of non‐Western settings, it is important to attempt to develop these.^9^ For instance, genome research requires diversity to increase the accuracy of disease prediction and ensure the quality of treatment for individuals from underrepresented communities.^7^ To explore the genetic basis of COVID‐19 variants of concern and the recombination of SARS‐CoV‐2 mutations, as well as the differences in disposition to the disease,^10^ understanding provenance is necessary—and this requires the investigation of clinical and other data from Africa, which is currently unavailable.^11^ To be successful in diversifying health data, requires a strong vision on the return from efforts for digital data capture for the quality of care in Africa.^8^

This investigation presents the findings of a design process for an inclusive data management system for health in Africa. The design problem studied here is a “wicked design problem,”^91^ in that the objectives of the data management system are only loosely defined and must take into account different stakeholders with different understandings of the problem to be solved, as well as the objectives of the data management system to be developed. Buchanan^13^ ^(p. 96)^ defines “design” as “the conception and planning of the artificial”, which, according to Rittel,^12^ is especially relevant to problems in mathematics that cannot be solved by linear models. In solving such problems, the definition of the problem and the problem solution need to be distinguished.

There is no objective or “right way” to frame such a problem, and there is usually more than one solution. In fact, the way the problem is seen depends heavily on the worldview of the designer.^12^ In order to address a problem, it first needs to be understood and all the relevant stakeholders considered. How the problem is framed depends on the perspective that is taken to describe and structure the problem, which determines the way the problem is handled— and the solution is envisaged.^14^ This is an iterative process, given that the “information needed to understand the problem depends upon one's idea for solving it”^12^ ^(p. 161)^. A key aspect of design thinking is that the creation and adaptation of a fruitful frame has been identified by all stakeholders involved.^15^, ^16^ The development of a frame for understanding the problem is also referred to as “pre‐development,” or the “fuzzy front end,” which is critical for successful innovation.^17^ A successful approach—suggested by Sanders' evolving map of design practice—would have the expert users react to the process, while the other participants function as co‐creators in the design process, thereby balancing an expert mindset with a participatory mindset.^18^

Kingdon^19^ explains how new ideas can emerge in a dynamic social context, differentiating between the way in which the problem is framed (problem stream), the solutions available for the resolution of a problem (policy stream), and the salience of the problem in the sphere of politics or political mood (political stream). New ideas may enter the public agenda when the streams come together, that is, when a policy window opens. Kingdon coined the term “policy entrepreneurs” to refer to those who help bring together one or more streams to put a policy on the agenda. In this process, historical junctures are identified, which are potentially critical, because they loosen the structures in place, allowing for the shaping of new things that diverge from the past.^20^ A critical juncture or turning point is a defining point in time that may narrow the path for a solution into a certain direction, or broaden it for the inclusion of new elements previously not considered.^20^ This article presents the findings of research on the extensibility of recently adopted guidelines for data management to improve data diversity in a network of worldwide interconnected data.

## 2 PRE‐DEVELOPMENT: UNDERSTANDING THE CONTEXT

For the framing of the problem at hand, the context needs to be explored. In this section, several issues are described that are relevant to the problem of health data management in Africa. These are: (a) lack of ownership of health data in Africa; (b) lack of health data diversity, resulting in less relevant data‐based solutions for Africa; (c) obstacles to the digitalization of health care in Africa; (d) monopolies and the commercial use of (health) data in Africa, and (e) differentiation in regulations with incompatible requirements across regions and continents. Tension over, and lack of, ownership of health data in Africa compounds the difficulties caused by the limited availability of data. This is holding back the global study of genomics data, among other things.^21^

On the African continent, data ownership is an ongoing concern,^22^ especially on leading global research topics such as genomics and virus pandemics. Data are valuable, so how data is obtained and who benefits from it, is a sensitive question. Participation in research is not an unusual approach taken in Africa to allow people to access medical aid, but this places these people in a vulnerable situation, as they are often asked to sign a form allowing researchers to control how their data will be used, for instance, in human genome studies.^21^ A group of African researchers found that this generates distrust between African people and Western researchers, as participants are required to sacrifice their autonomy to make their own decisions about data ownership.^23^ Concerns over lack of ownership of African data in health are most poignantly illustrated by the data on Ebola collected from Liberia, which is no longer available in their entirety in its Ministry of Health. Part of this data is now only findable through WHO‐facilitated situation reports.^24^ In line with these observations, the African Academy of Sciences Commission on Data and Biospecimen has identified consent, research integrity, data governance and access, ethical and regulatory oversight, and what is referred to as a poor “African negotiating position” over its data as contentious areas.^23^

Lack of ownership of health data in Africa undermines the “social contract” that “ensures the rights of the patient, considers the community's best interest, and prioritizes social value as a research objective”.^23^ The interests of research participants should be put first^23^, ^25^, ^26^ for a global emergent response to infectious disease outbreaks such as COVID‐19, for which a real‐time, broad‐based, continuous, and collaborative framework for data collection, sharing, analysis, and alerts is needed.^27^ Due to the unsystematic inclusion of data reported from Africa and fear of under‐reporting, the COVID‐19 crisis in Africa has been referred to as “the silent epidemic”.^28^, ^29^ Research by Imperial College London found that in Sudan only 2% of COVID deaths were reported in the capital Khartoum in 2020 and that there were 16 090 (95% CI: 14300‐17 990) undetected COVID‐19 deaths in the capital alone up to November 20, 2020.^30^ In addition, media‐reported evidence from other countries in Africa demonstrates severe under‐reporting, which is impacting on the global prioritization of COVID‐19 responses in Africa.^31^, ^33^ The lack of such data is also holding back surveillance on any genomic associations with COVID mutations.^34^, ^35^ Hence, while data is being heralded as the “new gold,”^36^ the collection, storage, and ownership of digital health data remains a contentious issue, especially as it is unclear how the African continent benefits from such data.

An increased interest in digital health, e‐health, and m‐health among actors outside the African continent^37^, ^38^, ^39^, ^40^ encounters many barriers on the ground.^41^ Despite a steady increase in domestic legislation on digital health in African countries (such as Kenya, Uganda, Ethiopia, and Zimbabwe, among others),^42^ Basajja^43^, ^44^ found that most clinics and hospitals in Uganda are still working with pen and paper. Moreover, these health facilities may be struggling with situations that the (Western) health system paradigm does not account for,^45^ as well as coping with different health orientations that co‐exist in communities.^46^ At a technical level, innovation is impeded by a lack of broadband,^39^ lack of integration and interoperability, the re‐use of parallel digital health data streams,^43^, ^47^ weak Internet security,^48^ limited access to power and power instability,^49^ the incompatibility of the technology used with the context of implementation,^50^ ageing equipment and lack of ability to sustain and/or expand it in the health sector,^51^ and lack of involvement of national research and development.^52^ Under such conditions, digital data is unlikely to improve the quality of care on the ground in Africa. These problems strongly affect the opportunities and challenges that computer science‐experts are facing when developing a computerized system of data management for health in the African context.

The monopolization and commercialization of digital data is another problem. The risk of loss of net neutrality due to the private upscaling of broadband (by Facebook, 2Africa cable, and Google, among others) compounds the problem of lack of data ownership in Africa.^29^ Lack of regulatory frameworks for data ownership and the monopolistic trends of U.S. platform companies, which have little interest in protecting the data subject, contribute to a loss of control over digital data and its processing on the African continent—despite the economic value that such data represent and the gains it could help the continent make.^1^ As an example, the current District Health Information System (DHIS), which is widely used on the African continent, facilitates health facilities to produce digital data, which are then uploaded to the system.^44^ However, the health facility itself does not generally have access to the data, or the analytics from it, and generally lacks the expertise to use such data to improve quality of care.^43^, ^47^

Regulatory policy about data ownership has seen a revolutionary change in the last decade. The European Union's (EU's) General Data Protection Regulation (GDPR)^53^ builds on previous European frameworks that deem the owner of data to be the data subject. China is expanding its regulatory framework for personal data protection in the area of privacy rules for companies.^54^ The different legal settings for data‐handling and data‐localization may exacerbate the division of the Internet into a Balkanized “splinternet” with incompatible requirements for access in different jurisdictions, which may threaten the global nature of the Internet, as originally perceived.^55^

All of the above issues pose constraints on the available structure for digital data‐driven health care and medical research, limiting the benefits to African stakeholders and restraining their interests. This is increasingly understood to be an issue at the heart of the problem affecting the COVID‐19 recovery. Leveraging digital transformation in the post‐COVID era is generating political interest in investing in digital health data‐based solutions, providing opportunities for youth entrepreneurship, responding to Africa's young demographic,^56^ while recognizing the challenges ahead in addressing the problem of inclusive digitization.^57^ Hence, a window of opportunity has opened to reassess the available models to manage, store, and collect data, especially health data, in Africa and other non‐Western geographies.^58^, ^59^

## 3 METHODOLOGY

The solution proposed here is to design an improved Health Data Management System (HDMS) for Africa using an ethnographic design in which stakeholders are identified and included in the development of the solution. The development team includes both computational experts and practitioners from the field. The objective of data management is to “provide analytical information to help drive operational decision‐making and strategic planning.”^60^ This research identifies the potential of alternative data management for health data in view of the agenda‐setting process, distinguishing the perception of the problem, the relevance of the problem as perceived by the political mood, and the solutions available to address the problem.

The research presented in this article covers 5 years (2016‐2021) and was carried out in two phases. In the first phase (2016‐2018), the principal investigator served as an advisor to the East Africa Health Research Commission (EAHRC).^61^ The Commission falls under the East African Community (EAC), whose members are: Burundi, Kenya, Rwanda, South Sudan, Tanzania, and Uganda. This is a political body and, therefore, provides a good platform from which to study the framing of the problem of digital data management in the context of agenda‐setting. During this phase, problems with health data management were identified, culminating in an agreement within the EAHRC on an integrated HDMS. During the second phase (2019‐2021), a research group was established, led by the principal investigator, in countries across the whole of Africa: Uganda, Ethiopia, Kenya, Liberia, Nigeria, Somalia, Tanzania, Tunisia, and Zimbabwe. The selection of countries was based on the availability of interest among researchers, capacity to engage, and relevance to the project. This research group, called the Virus Outbreak Data Network (VODAN)‐Africa, set as its objective the realization of a proof of concept of the design approved by the EAHRC.

## 4 FAIR DATA

FAIR data stands for data that is “Findable,” “Accessible” (under well‐defined conditions), “Interoperable,” and “Reusable.”^62^ The FAIR Principles were adopted in January 2020 for all data collected under EU research funding and applying to external geographies. Such data should be described in detail and is referred to as “metadata”—which is a set of data that gives information about other data in machine‐readable format. The “FAIRification” process is the production of metadata on provenance (using Dublin Core Terms) and content through machine‐readable vocabularies of data.^63^ “FAIRness” refers to the modular FAIR Principles, which provide for a spectrum of compliance. FAIRification can be implemented by workbenches such as the Stanford University CEDAR Workbench for Open Science and Bioportal, which self‐identifies as FAIR‐compliant,^64^ or semantic web‐based tools through linked data,^65^ such as DS Wizard, or Elixer 92. The potential to draw on FAIRified federated sources has attracted attention from the artificial intelligence (AI) community.^66^ The implementation of FAIR is supported by the GO FAIR Implementation Networks (IN). Recognizing the dynamic process of its construction,^58^ these networks consist of implementation communities in which FAIR Principles can be mutually developed and adapted to the needs of the group.^67^ The implementation of GO FAIR is structured around three activities: changing stakeholders (GO CHANGE), building FAIR technology (GO BUILD), and training participants to use FAIR (GO TRAIN).^68^

## 5 TURNING POINT 1: THE CONCEPT OF AN EAST AFRICAN OPEN SCIENCE CLOUD

**FIGURE 1:**
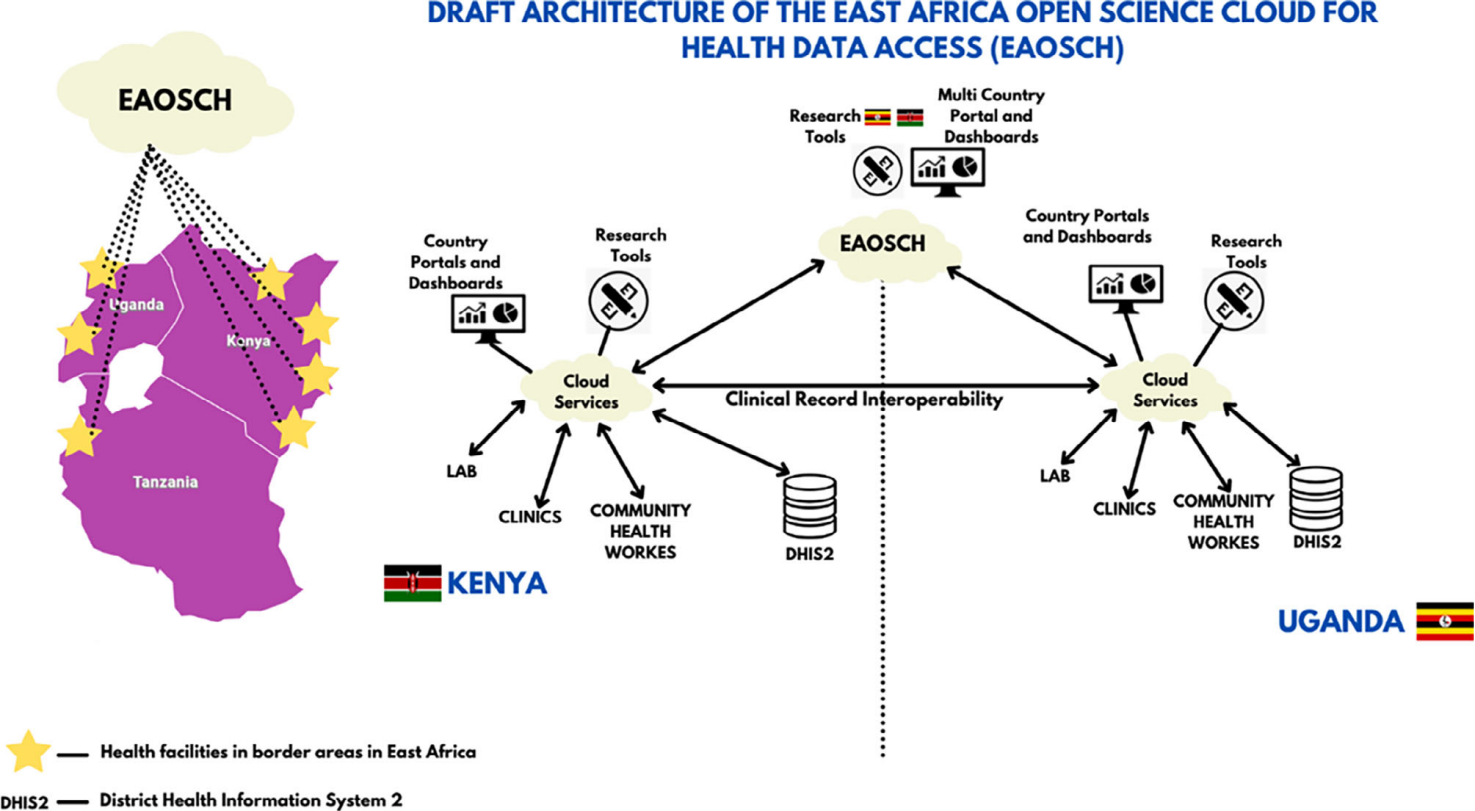
Original concept of an East African Open Science Cloud for Health (Mirjam van Reisen, Obinna Osigwe, 2021), based on architecture discussed in 2017 at the EAHRC Conference in Entebbe, Uganda, and presented in 2018, EAHRC Digital Health Conference, Kigali, Rwanda, by Hariet Nabudere^69^

The work in Africa started in 2016 with consultations in the EAHRC. During the preparatory phase of the East Africa Cross‐Border Health Integrated Partnership Project (CB‐HIPP), various aspects of data sharing across borders in different health jurisdictions were analyzed. In a series of meetings in 2016 and 2017, officials from the EAHRC, experts and practitioners formulated a common understanding and framing of the problem. In 2017, advisors associated with the EAHRC also attended a Lorentz FAIR implementation workshop at Leiden University, where the basic outline for an architecture was developed (see Figure 1). The design used machine‐readable metadata that could be kept in residence in health facilities, but could also be pushed from local facilities into national systems and the regional interface, providing a common dashboard across regions and bridging national jurisdictions. The data included patient data and research data.

In the EAOSCH design, presented in 2018, the anonymized records of patient data would be pushed to a health management information system (HMIS), which could be the DHIS2 (https://dhis2.org/), a patient record system commonly used in East Africa.^69^ This would be a new feature as the DHIS2 currently has limited (if any) analytical application in practice in the lower layers of health facilities and among health workers.^70^ Within the EAOSCH design, different layers of data were identified—raw data, anonymized data and aggregate data—but there was no explicit strategy for a machine‐readable semantic vocabulary. The data were exchanged between various locations via cellular networks, SMS or over the Internet. The EAOSCH design responded to the need felt by stakeholders for a more agile data analytical system, which would increase usability for planning within clinics and hospitals to enhance the quality of care, as well as monitoring, policy and planning by policymakers at the national, regional and international levels. The FAIR Principles were adopted as a way to attain this.^61^

The agreement on a FAIR‐based Open Science Cloud for Health as a foundational architecture can be regarded as the first turning point. This agreement created important new possibilities to reflect on the basic principles for health information systems. It also opened up the design to the possibility of FAIR‐based clinical data, accessible under well‐defined conditions, held in a distributed manner across different legal sovereignties. The design received the support of a broad range of experts from the East Africa partner states at a conference held in September 2017 and at subsequent stakeholders meetings. The architecture was eventually adopted by the EAC Sectoral Council of Ministers of Health in 2017.^61^

## 6 TURNING POINT 2: FEDERATED FAIR PRINCIPLES ADOPTED IN AFRICA FAIR COMMUNITIES

### 6.1 Agreement to adopt FAIR

The key element that emerged from the agreement adopted by the EAHRC on the EAOSCH was the need to develop a strategy for machine‐readable controlled vocabulary. This required a “community” to be set up, around which such vocabulary could be developed. The vehicle for this was the FAIR INs. Following the work of the EAHRC, FAIR IN‐Africa was established in 2019. FAIR IN‐Africa looked at the possibility of African universities and service providers being connected to the Internet of FAIR Data and Services (IFDS), which necessitated the investigation of the following^67^:

- The use of the FAIR Principles in helping solve the problem of the extraction of data from the African continent without returning benefits
- The possibility of a system of governance of data that would benefit the African continent
- The emerging possibility through FAIR of rooting digital data within an African philosophy, whereby data is owned by the data subject
- Exploration of the contribution that Africa philosophy can make to the global IFDS due to its epistemology of united and collective existence expressed within local realities
- Engagement with the FAIR initiative to strengthen an African connection, perspective and orientation in a globally connected world

In the context of IN‐Africa, data‐ownership was identified as a critical issue for any further design. It was considered that the federated structure of FAIR‐data could be a potential key feature to allow data to be curated “in residence”—that is, in the location where it is produced under the appropriate governance. Ad hoc distributed learning in the context of medical data, defined as learning from data without the data leaving the hospital, had been successfully implemented by Jochems et al in 2016.^71^ The potential of FAIR, machine‐readable and federated data, useable for AI, under the ownership of the data subject, forms the foundational idea for the design process.

FAIR IN‐Africa provided the basis for a research consortium and for building a workable architecture with suitable technological tooling. In FAIR language, this is referred: GO TRAIN, GO CHANGE, and GO BUILD. Following the establishment of FAIR IN‐Africa, in 2020, a pilot design study was started by VODAN Africa, implemented under FAIR IN‐Africa. VODAN Africa adopted FAIR for the implementation of a COVID‐19 data collection methodology with an explicit dedication towards federated data‐curation. The acceptance of federated FAIR Principles in February 2020 by the two FAIR communities in Africa (FAIR IN‐Africa and VODAN Africa), can be regarded as the second turning point, defining the future course of the design.

### 6.2 FAIR Equivalency in different jurisdictions

In order to advance the concept of federated digital health data in African sovereignties, the legal boundaries of digital health data processing in different jurisdictions was investigated, recognizing that different health data, policies and political climates are at play. An analysis was carried out, which resulted in an index referred as FAIR Equivalency. FAIR Equivalency is an indication of the degree of agreement between the national regulatory situation and the FAIR Principles.^42^ This index is based on the FAIR Principles (see Box 1) and can be categorized into four groups with underlying sub‐indicators, called FAIR facets. These are: “Findability” (F1, F2, F3, F4); “Accessibility” (A1, A1.1, A1.2, A2); “Interoperability” (I1, I2, I3) and “Reusability” (R1, R1.1, R1.2, R1.3).^62^

BOX 1 **FAIR Principles and FAIR Equivalence**

The facets of the FAIR Principles are as follows :

**Findable**

F1: (meta)data are assigned a globally unique and persistent identifier.

F2: data are described with rich metadata (defined by R1 below).

F3: metadata clearly and explicitly include the identifier of the data it describes.

F4: (meta)data are registered or indexed in a searchable resource.

**Accessible**

A1: (meta)data are retrievable by their identifier using a standardized communications protocol.

A1.1: the protocol is open, free, and universally implementable.

A1.2: the protocol allows for an authentication and authorization procedure, where necessary.

A2: metadata are accessible, even when the data are no longer available.

**Interoperable**

I1: (meta)data use a formal, accessible, shared, and broadly applicable language for knowledge representation.

I2: (meta)data use vocabularies that follow FAIR Principles.

I3: (meta)data include qualified references to other (meta)data.

**Reusable**

R1: meta(data) are richly described with a plurality of accurate and relevant attributes.

R1.1: (meta)data are released with a clear and accessible data usage license.

R1.2: (meta)data are associated with detailed provenance.

R1.3: (meta)data meet domain‐relevant community standards.

(FAIR principles: https://www.go‐fair.org/fair‐principles/)

As a first step, all of the regulatory and policy documents relevant to health and digital health in a jurisdiction are collected. Subsequently, the level of FAIR Equivalency is analyzed by comparing the content of the documents with the 15 FAIR facets. For this, a closed coding‐labeling approach was used in which the FAIR facets were compared with the corresponding statement in the policy document and given a score of correspondence. The FAIR Equivalency Score (FE‐Score) is the aggregate score of all of FAIR facets. This procedure was first carried out in Uganda^42^ and then in Indonesia,^72^ Zimbabwe,^73^ Nigeria,^74^ Ethiopia,^75^ and Kenya.^76^ The overall results of the FAIR Equivalency analysis was that there is adequate scope for the implementation of a FAIR‐based health architecture for the pilot study. This enabled a pilot to be established under the VODAN‐Africa research, which started in March 2020.

### 6.3 WHO SMART guidelines

A further push was given to the acceptance of such a framework in February 2021, when WHO launched its SMART Guidelines, that is, that data be standard‐based, machine‐readable, adaptive, requirements‐based, and testable.^77^ The VODAN Africa architecture conforms with all of the WHO SMART guidelines. WHO also specifically identifies the need for integrated data, with quality of care as the main objective. While the WHO design is one‐directional, VODAN Africa looks at the interoperability in a two‐way information stream. The WHO proposition demonstrates that the identification of a similar problem frame, may lead to a slightly different design. The SMART approach launched by WHO^77^ shows the political support at the global level for a new approach to create greater meaning in relation to digital data generated in health care. The linking of COVID‐19 data across borders and continents has generated interest among Asian countries in participating in the network,^78^ pointing to the likely viability of the concept across continents.

## 7 TURNING POINT 3: PROOF OF CONCEPT

The first task of the pilot phase towards a proof of concept of interoperability, based on federated data through data visiting, was to arrange partnerships for the deployment of FAIR Data Points within countries, a process that involved ministries and universities. In all participating countries, approval was arranged through the relevant authorities. A website was established to provide information on the project (https://www.vodan-totafrica.info/) and a regular reporting system established, including records of all sessions on YouTube (https://www.youtube.com/channel/UCbYaFxwAENKqEv3L1TUctgA), with a training of trainers phase (https://www.youtube.com/watch?v=Ei60DhGqcVE; VODAN Africa, 2020). The experience during this phase was that researchers acknowledged the problem, political concern about the issue of data ownership was high, and the approach was considered relevant in light of the regulatory settings and policy direction. In this phase, the key question was whether or not a solution was technically feasible, and whether or not a proof of concept could be achieved.

In keeping with the original architecture of the EAOSCH (Figure 1), the pilot phase started with two sets of data: clinical patient data and research data. The pilot started with data relevant to COVID‐19. The steps of the pilot phase were identified as: (a) testing of the proposition without an online realization; (b) presentation of a clear proposition to stakeholders; (c) implementation of FAIR Equivalency analyses (as per above); (d) approval by stakeholders in all locations; (e) establishment of 10 FAIR Data Points reachable over the Internet; (f) machine‐actionable data production (test data and real data); (g) running of queries over the Internet across the FAIR Data Points that visit the machine‐actionable clinical patient data; and (h) completion of successful proof of concept.

For the production of data, a human and machine‐readable WHO electronic COVID report form (e‐CRF) was prepared on an installable FAIR Data Point. Between July and September 2020, a total of 10 FAIR Data Points were installed across the African continent. The machine‐actionable FAIR Data Points were visible and reachable on the Internet, calling home to the VODAN FAIR Data Point community, meaning that they were findable by algorithms run over the Internet.^79^ Once this was achieved, the proof of concept test was carried out to run queries on the FAIR Data Point of Kampala International University and at the Leiden University Medical Centre. This was successfully realized, when queries were run across the two continents in September 2020 and federated data was computed^80^ (see Box 2). The successful proof of concept confirmed that implementation of the FAIR Principles for observable patient records in health facilities was a relevant approach to increasing access to reliable observational health data.^80^

BOX 2 **VODAN Africa FAIR data project milestones**

1. Successful cooperation and communication was achieved for increasing reliable health data:

- In the curation of clinical health data compliant with the regulatory framework in geographies where data is produced
- By ensuring that data ownership remains within the place where data is produced, increasing understanding of data provenance
- By creating the conditions for compliance with the EU GDPR
- Federated data curation strengthens the contextual nature and existence of the data allowing for the exploration of contextual variables of such data.

2. Required changes in approach to machine‐readable data production:

- Flexibility on machine‐readable data production is needed to (a) create interoperability of data with different organizational or content structures and (b) integrate data with different domain origin, such as clinical observational data and research data.

3. Future sustainability of the VODAN Africa FAIR data project depends on:

- Ensuring that data production increases understanding of the relevance of such data at point of care and improves the quality of health
- One‐directional use of the data (away from where the data is produced) being replaced by a model of collaboration with a view to benefiting the data subjects
- The capacity for data stewardship for data handling, processing, analytics and visualization being enhanced to service the data producers^80^

With regards to the FAIR metadata production of research data, the proof of concept did not succeed. While relevant research data were collected on the incidence of COVID‐19 among marginalized refugee and migrant communities in Tunisia, the limited scope of the WHO e‐CRF did not allow for the production of the data itself in a machine‐readable format and the team had to revert to traditional forms of data analysis. This was a major setback for the team and for the objective of integrating both patient data and research data. The conclusion was that a different approach was required (see Box 2).

## 8 TURNING POINT 4: CONSENSUS ON THE VODAN AFRICA ARCHITECTURE

To be sustainably deployed in the field, the perceptions of health workers and medical professionals need to be understood in order to facilitate the GO BUILD aspect of GO FAIR. In this regard, a study into information flows in clinics showed a large discrepancy between assumptions about the situation on the ground and the real life situation.^43^ The issues raised during the assessment highlighted the need for: (a) flexible and agile machine‐readable data production, and templates to be seamless related to the data flows in clinics and hospitals; (b) flexible and agile machine‐readable data production for research data, convergent with the controlled vocabulary used in the community; (c) tooling that would allow the production of bulk data production; (d) export capability of the produced metadata in the HMIS to avoid work duplication in facilities; (e) a clearly defined access and control architecture; and (f) the agile integration of observational patient data and research data within a controlled community.^81^

The conclusion was reached that sustainability would depend on the ability to increase data analytical understanding within clinics and hospitals and that, for this, further capacity building was needed (see Box 2). The research team agreed that for the next development phase a requirements and specifications exercise would be carried out to ensure that the future direction would match the conditions on the ground.^82^ In preparation for the next stage, the requirements and specifications for the tools were identified so that these would support the information architecture within the health facilities (see Box 3).

BOX 3 **Decision points and turning points**

**Design**

D1: Political adoption of a regional FAIR‐based East Africa Open Science Cloud for Health in 2017.

D2: Adoption of FAIR Principles for federated deployment by the FAIR IN‐Africa.

D3: Compliance with regulatory frameworks.

**Tooling**

Requirements

R1: Flexible human and machine‐readable data production (based on VODAN controlled vocabulary).

R2: Localization of the metadata system.

R3: Bulk input of data in data production platform.

R4: Usability and demonstration of value.

**Specifications**

S1: Open source.

S2: Programmability and adaptability.

S3: Own maintenance.

S4: Availability for training.

S5: Convergence with other FAIR developers to increase efficiency.

**Turning points**

T1: East Africa Open Science Cloud for Health in 2017 establishes the need for a within and across border health data sharing.

T2: FAIR IN‐Africa adopts FAIR Principles for federated deployment and increased data ownership in Africa.

T3: VODAN Africa team demonstrates data visiting works across two countries and two continents, September 2020.

T4: VODAN Africa team reaches consensus on the requirements and specifications for the clinical health data architecture in February 2021.

**Changes, redesign and deployment**

C1: Design needs to be radically adapted in order to fit realities of places deployed.

C2: CEDAR as a workbench to produce machine‐readable vocabularies.

R1: Flexible data production (based on VODAN controlled vocabulary).

**FIGURE 2:**
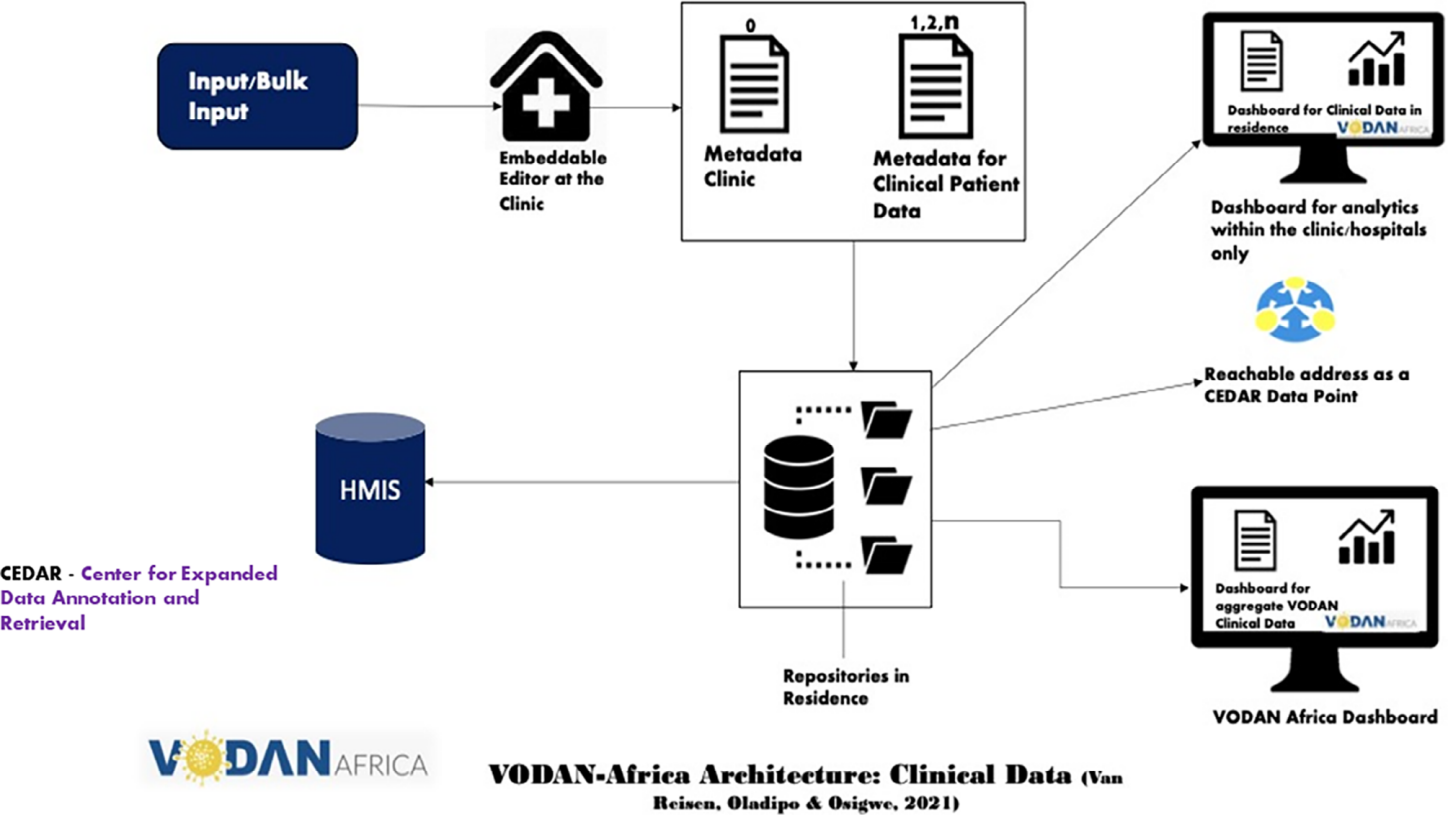
VODAN Africa architecture clinical data^83^

R2: Localization of the CEDAR Metadata System (Figure 2) in order to achieve:

- Convergence between CEDAR localized formats
- Localized availability of CEDAR templates for premise installation in 70 hospitals each in Uganda, Ethiopia, Kenya, Liberia, Somalia, Tanzania, Nigeria, and Zimbabwe (Figure 2)
- CEDAR templates based on the HMIS (including DHIS2) forms in use in the hospitals and with a VODAN agreed vocabulary (Figure 2)

R3: Bulk input of data in CEDAR platform (Figure 2).

R4: Usability and demonstration of value

**FIGURE 3:**
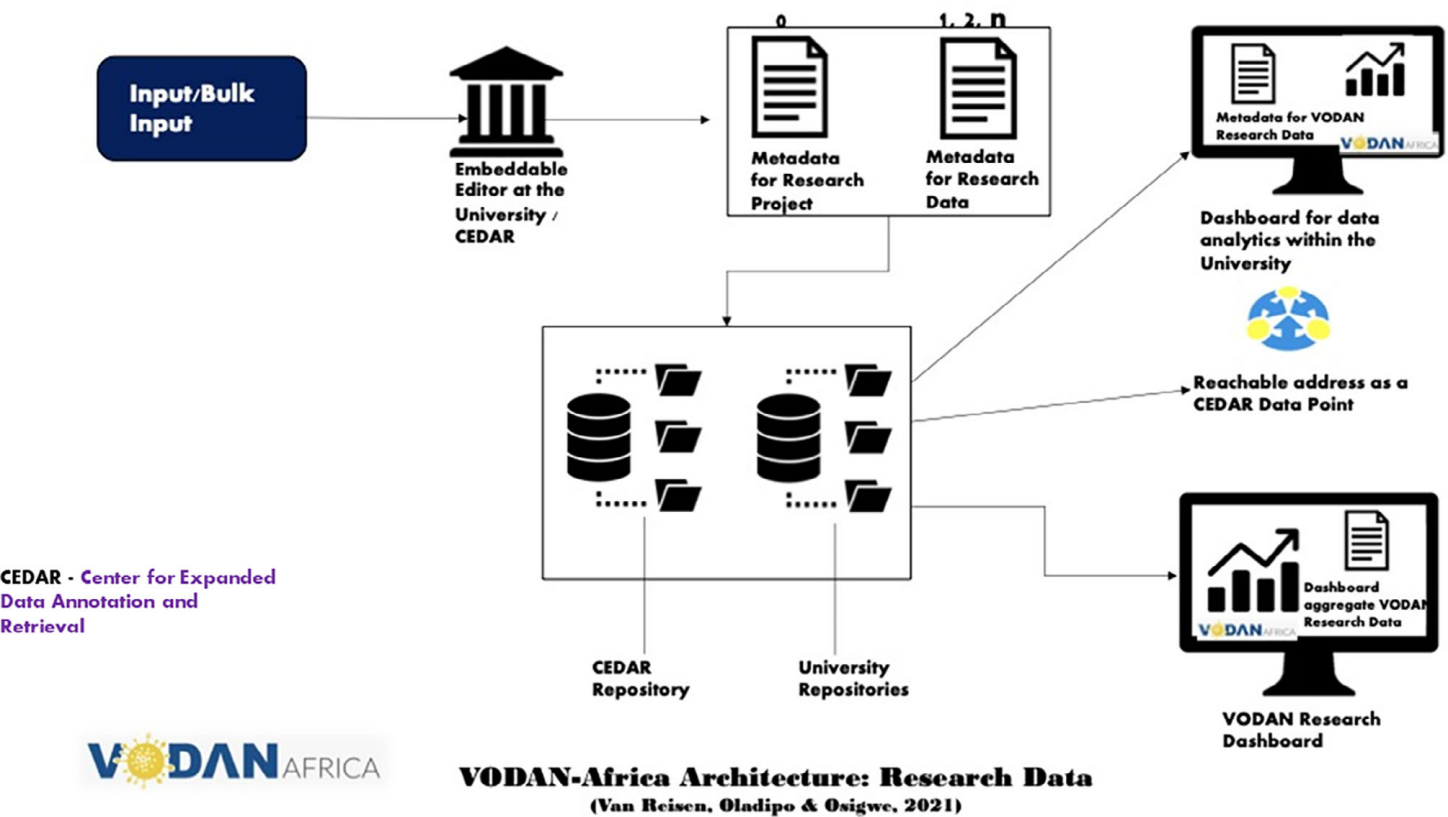
VODAN Africa architecture research data^83^

**FIGURE 4:**
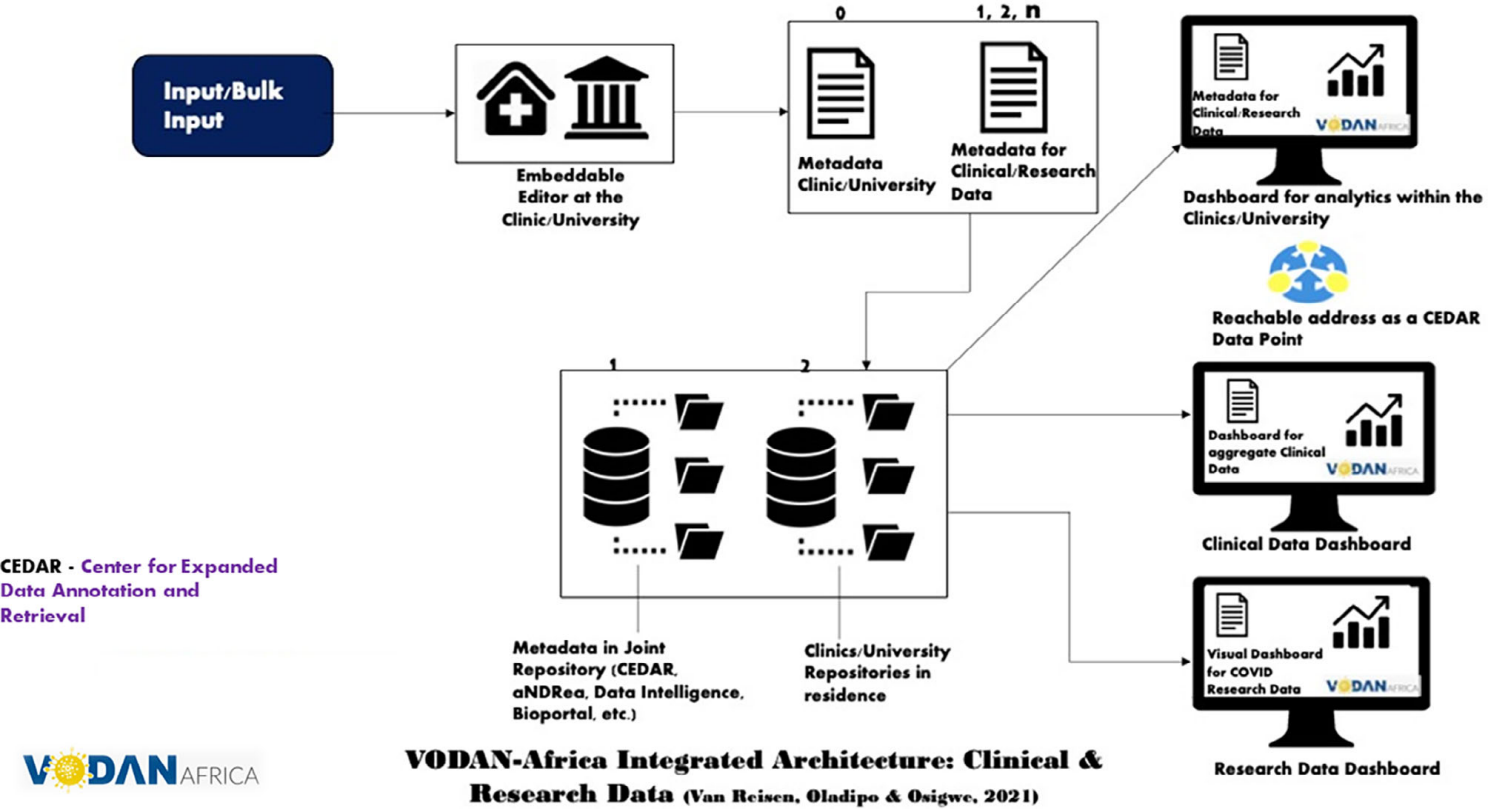
VODAN Africa integrated architecture clinical and research data^83^

**FIGURE 5:**
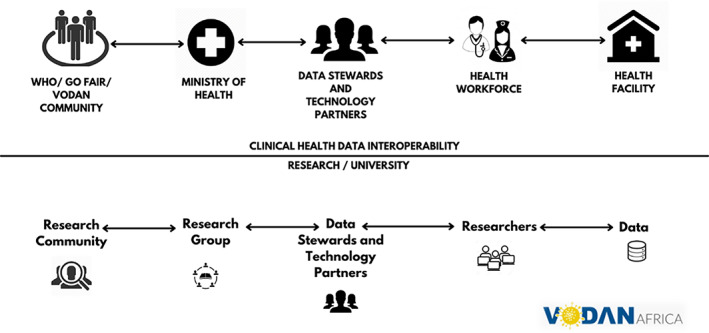
Organization of clinical health data interoperability with research communities^83^

- Human and machine‐readable data storage in a hospital‐controlled environment in Africa, with metadata pointing to the data in residence: own data repositories for hospitals are required
- Programming a tool for the transfer of the data included in the CEDAR templates into the HMIS/DHIS forms that hospitals can upload as per ministry regulations (hospitals do not need to input data twice)
- Ability to run queries within hospitals, between different in‐country hospitals and intercontinental
- Training for template development with controlled vocabularies of the VODAN Africa community
- Enabling of African data stewards to deploy across each of the implementation countries and partner hospitals in the other countries for visualization in dashboard format (within clinics and as VODAN Africa)
- Enabling of research‐data on COVID‐19 incidence to be published as human‐ and machine readable data, interoperable with VODAN Africa vocabulary in CEDAR and BioPortal **(**Figure 3 **)**
- Creation of synergy across FAIR leading projects **(**Figures 4 and 5 **)**

D1: FAIR Equivalency tool to measure FAIR compliance.

D2: Deployment of 10 FAIR Data Points based on DS Wizard.

D3: Data visiting by SPARQL Query across two continents through two facilities based on WHO eCRF.

D4: Bulk‐input into CEDAR.

D5: Localized embeddable editor for production of machine‐readable data on CEDAR.

D6: Localized bioportal installed to support local data production.

D7: CEDAR templates for outpatient registration in health facilities.

D8: Technical option available for output to DHIS2.

D9: Testing of off‐line functioning of localized CEDAR editor.

D10: Online course on FAIR data management for capacity development.

D11: Consensus on architecture for deployment.

Two systems were identified for testing: the Data Stewardship (DS) Wizard, which had developed the original WHO e‐CRF system and FAIR Data Points, and CEDAR. It was concluded that the best match with the requirements and specifications was offered by CEDAR. This can be considered the fourth turning point, and defines the next design phase, with the key elements identified as critical for deployment set out in Box 3. The CEDAR platform allowed for a flexible and superior production of machine‐readable data, however, it did not initially comply with several of the criteria. In a collaboration between the CEDAR platform and VODAN Africa, the following adaptations were programmed in Open Source, tested in real‐life context, including in entirely off‐line areas, and prepared for deployment:

- Bulk input of data in CEDAR templates
- Localized production of semantic machine‐readable data with a local embeddable editor bioportal instance
- Capture of metadata in local repositories
- Output of data to a HMIS

It was decided that instead of an open query capability, a closed dashboard would be more manageable, especially considering the fact that the certification of data points and algorithmic queries was not in place and that an open query capability would lead to fears and concerns within the health facilities about the protection of sensitive clinical patient data. The architecture for clinical patient data identifies the possibility of bulk input into a localized editor that is embedded in the health facility and through which two levels of data is produced in machine‐readable metadata: the clinic specification and the clinical patient data. These are stored as RDF and JSON linked languages in a local repository for data capture within the clinic or under the strict control of the facility, and preferably within the country. The repository has the capacity to export the data to DHIS2. The repository is identifiable through a reachable address on the Internet that can be indexed by Google. The usability of the data is arranged at two levels: the dashboard within the clinic and the aggregate dashboard of the VODAN community, creating real‐time data, resulting in the architecture in Figure 2.^9^, ^83^

The architecture for the research data is similar, with the inclusion of a repository within the university, which allows the data to remain in residence and creates a strong localized identity for the data (Figure 3). This strengthens the provenance of the data and adds meaning to it. For the research data, it is necessary that metadata are extensive and specific. Where templates are not available in a local embeddable editor, these can be constructed from the CEDAR platform.^84^

The combined clinical and research data leads to an aggregate dashboard for COVID‐19 data that is based on all data in the VODAN community.

When this architecture was realized, the community consisted of some 40 data stewards, health practitioners, academics, and people working in health policy.

The consensus on this architecture constitutes another turning point—a policy window in which the framing of the problem and the solution have been brought together with the political stream, which was already activated by the trajectory in the EAHRC. The team also realized machine‐learning analytical observations, based on the COVID‐19 FAIR‐metadata^85^ (see Box 3).

## 9 OWNERSHIP OF FAIR DATA IN AFRICA

Ownership of data is not only a matter of where the data is stored, but also which data or metadata to share (or not to share). Aligned with the GDPR, the VODAN Africa community unequivocally identifies data as belonging to the data subject. The privacy of personal data is non‐negotiable. The exposure of data is a decision that belongs to the data subject (see Box 3).

**FIGURE 6:**
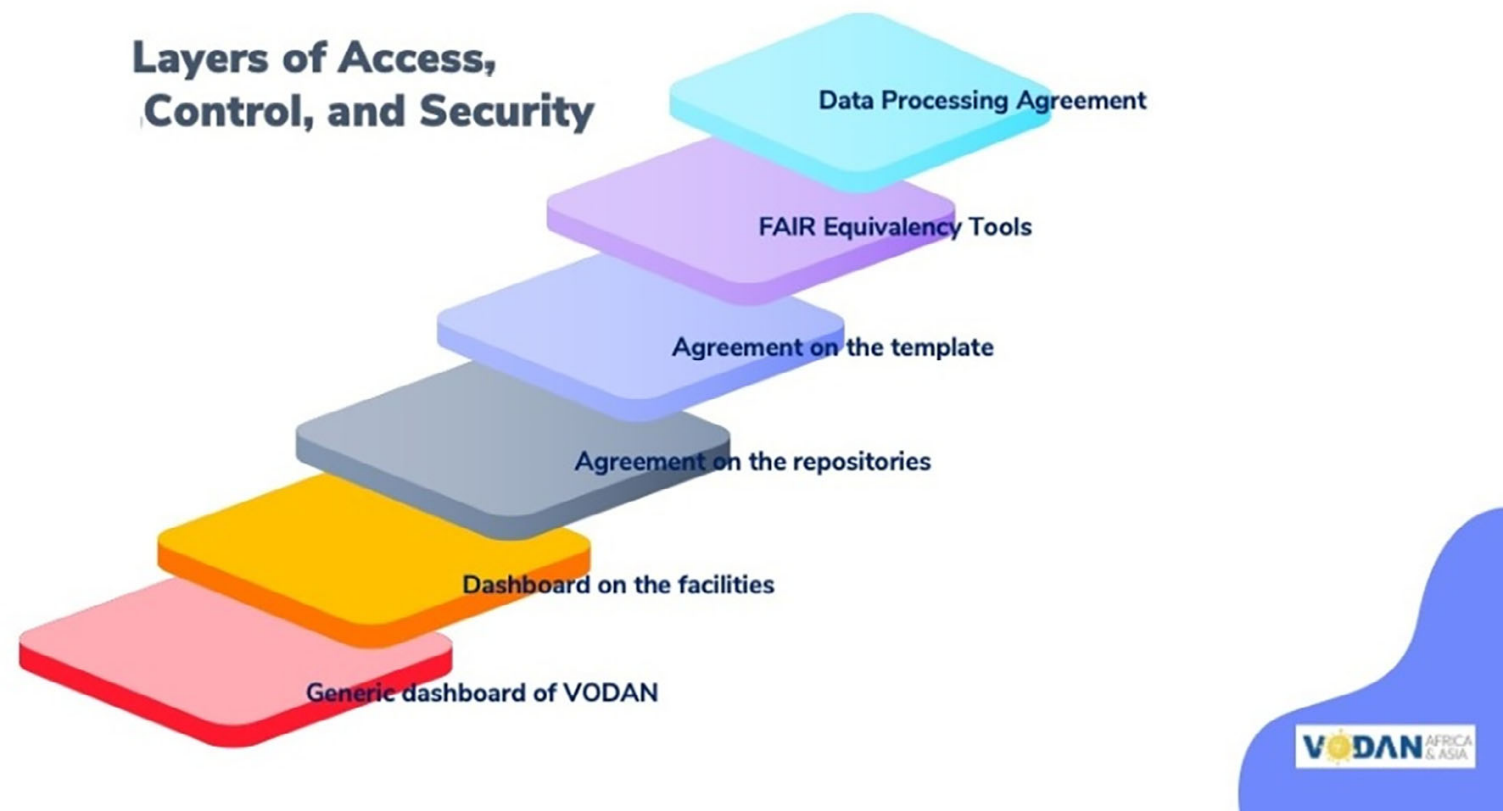
Layers of access, control and security of FAIR COVID‐19 clinical and research data

For patients, clinics, and authorities, a trusted environment is needed that sets clear rules about access to data, control, and security. In order to put in place a trustworthy set of layers, the following have been identified as necessary by the VODAN Africa community: (a) a data processing agreement, based on a joint and common agreement among all data processing partners, encompassing the strictest data control and processing requirements and equivalent to the GDPR; (b) the FAIR Equivalency tool to analyze specific areas of attention or opt out based on the regulatory framework in a given jurisdiction; (c) agreements about machine‐readable templates that are based on commonly used templates in the HMIS, such as DHIS (for clinical data only); (d) an agreement on repositories and levels of data security to keep data safe within the residence (repository) where the data is stored; (e) an agreement on data pipelines used for dashboards in the facilities providing key real‐time aggregate data at the facility level; and (e) an agreement on the data pipelines used for the VODAN dashboard to create aggregate information (Figure 6).

For the further development of machine learning/AI tools, the pipelines provided through the VODAN Africa dashboard will provide the most trustworthy way to enhance distributed capabilities.

## 10 CAPACITY BUILDING (GO TRAIN)

Building local research capacity is critical for the promotion of community participation, which will result in benefits to the community in terms of improved quality health care, requiring digital literacy support.^22^, ^86^ The design process described thus far has resulted in a dashboard for clinics and hospitals to assist with data collection and a data management system that is FAIR and fits within the boundaries set by the jurisdictions of the countries involved. Capacity building (GO TRAIN) is the final and critical pillar to realize FAIR data handling. In order to build the capacity to sustain such an architecture in Africa, the research group developed an online curriculum on a Digital Innovation and Skills Hub (DISH), which is a learning platform on FAIR‐based data science. Kampala International University was authorized by the National Council for Higher Education to offer digital lectures as a measure to counter the spread of COVID‐19. The COVID‐19 pandemic has been a catalyst for online learning, which has the potential to advance the inclusion of difficult‐to‐reach students within new areas of computer science and data science, including machine‐readable vocabularies, speeding up innovation in these areas.^87^, ^88^, ^89^ One of the courses on offer specifically teaches students about “Big Data and how to manage them [as well as] about the cutting edge of data and making data Findable, Accessible, Interoperable, and Reusable (FAIR).”^90^ This training prepares students to be data stewards.

## 11 CONCLUSION

The lack of data ownership on the African continent is a burden that has led to the under‐representation of Africa in global health data. In particular, it has impeded our understanding of the genetic basis of COVID‐19 and how the virus mutates across different populations, affecting control, prevention, response, and preparedness in Africa and globally. In addition, lack of interoperability and reuse of data in parallel digital health structures undermines the value of digital data health solutions, while the commercial use of such data creates distrust, particularly as there is little, if any, benefit derived from the collection of such data at point of care. The different regulatory frameworks for data capture and handling require a renewed vision on how to conduct data analysis across continents.

This study used an ethnographic design, including different stakeholders, to show that the Health Data Management Systems in Africa currently lack the capability and the ownership of data handling at the clinic level to strengthen data‐driven quality of care. A more meaningful process of data‐capture that focuses on the benefits at point of care could incentivize quality digital health data and contribute to solving the under‐representation of health data from Africa. The study focused on designing a data‐architecture that enables data capture for research in these domains. The investigation was conducted in two phases: first, within the context of a trajectory that led to the approval of an East Africa Open Science Cloud for Health in 2017; second, by the establishment of FAIR IN‐Africa in 2019 and, subsequently, by the design process of the VODAN Africa research team. The VODAN Africa team studied the possibility of a distributed architecture of linked human‐ and machine‐readable data held in residence or under strict control of the facility producing it.

The study identified four critical junctures or turning points that set the direction of the design:

- **Turning point 1**: East Africa Open Science Cloud for Health in 2017 establishes the need for a within and across border health data sharing
- **Turning point 2**: FAIR IN‐Africa adopts FAIR Principles for federated deployment and increased data ownership in Africa in 2019
- **Turning point 3**: VODAN Africa team demonstrates data visiting works across two countries and two continents in September 2020
- **Turning point 4**: VODAN Africa team reaches consensus on the requirements and specifications for the clinical health data architecture in February 2021

The first turning point was the political adoption of the East Africa Open Science Cloud for Health in 2017, emphasizing the need for health data interoperability within and across the countries in the region in a safe way. The second critical juncture was the adoption of the federated FAIR Principles within the community FAIR IN‐Africa, which set out to develop an architecture based on semantic, linked data held in residence, to facilitate data ownership. The third critical juncture was the proof of concept developed by the VODAN Africa team, which was carried out by the Leiden University Medical Centre and Kampala International University, and showed that the concept of data visiting works across two continents. The proof of concept also showed that the design needed to be radically adapted to fit the realities of the places where it is to be deployed. The fourth turning point was the selection of CEDAR as a flexible and agile workbench to produce machine‐readable vocabularies, adapted to local data production and for the data capture of repositories controlled by the facility that produces the data. The result is the VODAN‐Africa architecture in which clinical and research data held in residence can be visited across continents and real‐time information is available within health clinics, improving the availability of African health data.

The VODAN‐Africa team developed a tool to measure the convergence between the FAIR Principles and the regulatory framework in the different countries. This measure is called FAIR Equivalency and was applied in six countries, showing good compliance with national frameworks for digital health. The recently published WHO SMART Guidelines for (national) health data handling equally converges with FAIR Principles. These principles are also broadly compliant with the EU's GDPR, both concerning health and scientific data. The interest from other continents, notably Asia, shows there is a readiness to explore how data held in different regulatory frameworks can be shared for jointly agreed purposes of aggregate analytics, on the basis of distributed computational data visiting. This provides a solution to overcome the “splinternet,” while recognizing the diversity in regulatory frameworks for data control and handling across different jurisdictions.

A proof of concept was successfully carried out using a simple algorithm, that ran across two participating facilities on two continents, to visit the distributed data and produced aggregate findings. This showed that the design is developing into a mature proposition. The consensus on the VODAN Africa architecture across stakeholders in nine African countries provides the basis for a practical “Minimal Viable Product.” While it is important to keep data locally, visiting across borders and continents is possible. Most importantly, the distributed data, enhanced with metadata, will increase the quality of the data due to their rich metadata descriptions. The maintenance of provenance in the proposed solution is a critical feature that increases the situational meaning of data. The architecture that has been developed emphasizes the importance of local data production and analytical capabilities within health facilities and the need to invest in education to create the knowledge and confidence to innovate. The architecture is equally employable for clinical patient data and research data, which can potentially create a matching of information from different kinds of data including research data.

In sensitive research areas, such as genome research for COVID‐19, the participation of the African continent will be dependent on trust. Digital literacy and capacity to understand and maintain semantic web architectures is critical to sustain trust in this innovation. The architecture has the potential to be trustworthy, given the high priority given to African ownership, data‐use and capacity building. FAIR data in Africa will set a framework for responsible data, that acknowledges the right of the continent to manage, analyze and innovate its digital capabilities, and recognizes that Africa should own its own data, which, after all, is the new gold.

## CONFLICT OF INTERESTS

All authors declare they have no competing interests.

## AUTHOR CONTRIBUTIONS

**Mirjam van Reisen:** Conceptualization; funding acquisition; investigation; methodology; project administration; supervision; writing‐original draft; writing‐review & editing. **Francisca Oladipo:** Conceptualization; project administration; data curation; investigation; methodology; supervision. **Mia Stokmans:** Methodology; validation; Writing‐original draft. **Mouhamed Mpezamihgo:** Conceptualization; supervision. **Sakinat Folorunso:** Conceptualization; data curation; investigation; supervision. **Erik Schultes:** Conceptualization; formal analysis; validation. **Mariam Basajja:** Conceptualization; investigation; methodology; writing‐original draft. **Aliya Aktau:** Conceptualization; investigation; supervision. **Samson Yohannes Amare:** Conceptualization; data curation; formal analysis; investigation; methodology; software; supervision; validation. **Getu Tadele Taye:** Conceptualization; data curation; formal analysis; investigation; methodology; software; supervision; validation. **Putu Hadi Purnama Jati:** Conceptualization; data curation; formal analysis; investigation; methodology; software; supervision; validation. **Kudakwashe Chindoza:** Conceptualization; investigation. **Morgane Wirtz:** Conceptualization; data curation; formal analysis; investigation; supervision. **Meriem Ghardallou:** Conceptualization; data curation; formal analysis; investigation; methodology; supervision; validation. **Gertjan van Stam:** Conceptualization; formal analysis; investigation; methodology. **Wondimu Ayele:** Conceptualization; investigation; supervision. **Reginald Nalugala:** Conceptualization; investigation; supervision; validation. **Ibrahim Abdullahi:** Investigation; supervision. **Obinna Osigwe:** Writing‐original draft: design of figures. **John Graybeal:** Investigation; methodology; software. **Araya Abrha Medhanyie:** Conceptualization; formal analysis; investigation. **Abdullahi Abubakar Kawu:** Conceptualization; investigation. **Fenghong Liu:** Writing‐review & editing. **Katy Wolstencroft:** Validation; writing‐review & editing. **Erik Flikkenschild:** Validation. **Yi Lin:** Writing‐original draft. **Joëlle Stocker:** Writing‐review & editing. **Mark A. Musen:** Project administration; supervision.

### PEER REVIEW

The peer review history for this article is available at https://publons.com/publon/10.1002/ggn2.10050.

## ETHICS STATEMENT

Tilburg University, Research Ethics and Data Management Committee of Tilburg School of Humanities and Digital Sciences REDC#2020/013, June 1, 2020‐May 31, 2024 on Social Dynamics of Digital Innovation in remote non‐western communities.

Uganda National Council for Science and Technology, Reference IS18ES, July 23, 2019‐July 23, 2023.

Letter of Endorsement by the Government of the National Regional State of Tigray, Bureau of Health. Ethiopia, SAS/277/2020, October 7, 2020.

Data Processing Agreement between Kampala International University and Great Zimbabwe, Andrew Chindanya, Provost Chancellor, University, October 30, 2020.

Data Processing Agreement between Kampala International University and Musa Ango Abdullahi, Registrar, IBBUL (Nigeria), November 9, 2020.

Data Processing Agreement between Kampala International University and Dr Reginald Nalugala, Tangaza University College (Kenya), November 4, 2020.

Data Processing Agreement between Kampala International University and Dr Sakinat Folorunso, OOU (Nigeria), November 8, 2020.

Data Processing Agreement between Kampala International University and Prof Meriem Ghardallou, Université de Sousse (Tunisia), November 9, 2020.

Data Processing Agreement between Kampala International University and Addis Ababa University, Dr Wondimu Ayele (Ethopia), November 10, 2020.

Data Processing Agreement between Kampala International University and Mekelle University, Dr Araya Medhanie (Ethopia), February 3, 2020.

Data Processing Agreement between Kampala International University and East Africa University, Dr Jamal Mohamed Warsame (Somalia), February 3, 2021.

## Supporting information

Supplementary TPR File

## Data Availability

The data that support the findings of this study are openly available in OSF at https://osf.io/q49wm/ Reference: IN‐Africa and Ambassadors The data that support the findings of this study are openly available at: https://bioportal.bioontology.org/accounts/vodana Ontoportal: https://ontoportal.org/ VODAN published as nano publication: http://server.nanopubs.lod.labs.vu.nl/RAdDKjIGPt_2mE9oJtB3YQX6wGGdCC8ZWpkxEIoHsxOjE http://server.nanopubs.lod.labs.vu.nl/RAh2Ce_5uDb6qBRuGTriAx9Es6WQxmVtaeCPEAclOCbU4#VODAN VODAN FIP: https://osf.io/gz2st/ The VODAN FIP here used as the basis of the ZonMw COVID Program FIP: https://fip-wizard.ds-wizard.org/projects/8c681c55-3dc8-42eb-93dd-8b27cf40bcc3/documents CEDAR revised case report form for Confirmed Novel Coronavirus COVID‐19: https://metadatacenter.org/ https://openview.metadatacenter.org/templates/https:%2F%2Frepo.metadatacenter.org%2Ftemplates%2F3dd62fd4-3e77-414f-b5f5-01d25acf54f6 Basis for dashboards (based on java/react): https://dcw.metadatacenter.org/ All code in Open Source and available in: https://github.com/VODANA The data that support the findings of this study are openly available at: [https://www.vodan-totafrica.info/], https://www.vodan-totafrica.info/webinar-series.php?i=1&n=cedar-localisation The data that support the findings of this study are openly available at: https://www.go-fair.org/ https://www.go-fair.org/implementation-networks/ https://www.go‐fair.org/implementation‐networks/overview/vodan/ https://www.youtube.com/channel/UCbYaFxwAENKqEv3L1TUctgA FAIR data management Distance Learning Course, KIU: https://lms.kiu.ac.ug/course/index.php?categoryid=279 https://lms.kiu.ac.ug/course/view.php?id=3888 (access permission needed).

## References

[1] United Nations Conference on Trade and Development . *Digital economy report 2019: value creation and capture—implications for developing countries*; 2019.

[2] Araujo D, Davids K, Passos P. Ecological validity, representative design, and correspondence between experimental task constraints and behavioral setting: comment on Rogers, Kadar, and Costall (2005). Ecol Psychol. 2007;19:69‐78. 10.1080/10407410709336951.

[3] Brunswik E. Distal focussing of perception: size‐constancy in a representative sample of situations. Psychol Monogr. 1944;56(1):i‐49. 10.1037/h0093505.

[4] Bronfenbrenner U. Toward an experimental ecology of human development. Am Psychol. 1977;32(7):513‐531. 10.1037/0003-066X.32.7.513.

[5] Abubakar A, de Vijver F. Cross‐cultural comparisons. Econ Bot. 2008;62(3):213.

[6] Diehl M, Wahl H‐W, Freund A. Ecological validity as a key feature of external validity in research on human development. Res Human Dev. 2017;14(3):177‐181. 10.1080/15427609.2017.1340053. PMC10978015 38550389

[7] Sirugo G, Williams SM, Tishkoff SA. The missing diversity in human genetic studies. Cell. 2019;177(1):26‐31. 10.1016/j.cell.2019.02.048. 30901543 PMC7380073

[8] Munung NS, Mayosi BM, de Vries J. Genomics research in Africa and its impact on global health: insights from African researchers. Global Health Epidemiol Genomics. 2018;3:e12. 10.1017/gheg.2018.3. PMC6152488 30263136

[9] Beyan O, Choudhury A, van Soest J, et al. Distributed analytics on sensitive medical data: the personal health train. Data Intell. 2020;2(1–2):96‐107. 10.1162/dint/_a/_00032.

[10] Sze S, Pan D, Nevill CR, et al. Ethnicity and clinical outcomes in COVID‐19: a systematic review and meta‐analysis. EClin Med. 2020;29:100630. 10.1016/j.eclinm.2020.100630. PMC7658622 33200120

[11] Mbow M, Lell B, Jochems SP, et al. COVID‐19 in Africa: dampening the storm? Science. 2020;369(6504):624‐626. 10.1126/science.abd3902. 32764055

[12] Rittel HWJ, Webber MM. Dilemmas in a general theory of planning. Policy Sci. 1973;4(2):155‐169. 10.1007/BF01405730.

[13] Buchanan R. Wicked problems thinking in design. Des Issues. 1992;8(2):5‐21.

[14] Dorst K. The core of ‘design thinking’ and its application. Design Stud. 2011;32(6):521‐532. 10.1016/j.destud.2011.07.006.

[15] Björklund TA. Initial mental representations of design problems: differences between experts and novices. Design Stud. 2013;34(2):135‐160. 10.1016/j.destud.2012.08.005.

[16] Dorst K. Frame creation and design in the expanded field. J Design Econ Innov Issue. 2015;1:22‐33. 10.1016/j.sheji.2015.07.003.

[17] Herstatt C, Verworn B. The ‘fuzzy front end’ of innovation. Bringing Technology and Innovation into the Boardroom. London: Palgrave Macmillan; 2004:347‐372. 10.1057/9780230512771_16.

[18] Sanders L. On modeling: an evolving map of design practice and design research. Interactions. 2008;15(6):13‐17. 10.1145/1409040.1409043.

[19] Kingdon JW. Agendas, Alternatives, and Public Policies. New York: HarperCollins Publishers; 1984.

[20] Soifer HD. The causal logic of critical junctures. Comp Pol Stud. 2012;45(12):1572‐1597. 10.1177/0010414012463902.

[21] Nordling L. Give African research participants more say in genomic data, say scientists. *Nature*; 2021. https://www.nature.com/articles/d41586-021-00400-9. Accessed May 13, 2021. 10.1038/d41586-021-00400-9 33597774

[22] Mawere M, van Stam G. eLearning in an African place: how ‘alien’ elearning models are failing many in Africa. *Information and Communication Technologies for Development. Strengthening Southern‐Driven Cooperation as a Catalyst for ICT4D*, 15th IFIP WG 9.4 International Conference on Social Implications of Computers in Developing Countries, ICT4D 2019, Dar es Salaam, Tanzania, May 1–3, 2019, Proceedings, Part II, 2019, pp. 421–432.

[23] Alliance for Accelerating Excellence in Science in Africa (AESA) . *Recommendations for data and biospecimen governance in Africa*, ASP Policy Paper 3, African Academy of Sciences; 2021. https://www.aasciences.africa/sites/default/files/Publications/Recommendations%20for%20Data%20and%20Biospecimen%20Governance%20in%20Africa.pdf. Accessed May 13, 2021.

[24] World Health Organization . Ebola data and statistics: Situation summary Latest available situation summary [online]. World Health Organization; 2016. https://apps.who.int/gho/data/view.ebola-sitrep.ebola-summary-latest?lang=en. Accessed May 13, 2021.

[25] Charity M, Nkwo M, Orji R, Ebere E. Personalized persuasive technology for maternal healthcare in Nigeria. Paper presented at: Persuasive Technology Conference, Aalborg, Denmark; 2020. file:///C:/Users/Susan/AppData/Local/Temp/4_ppt_nkwo.pdf. Accessed May 13, 2021.

[26] Nkwo M, Orji R, John U. Insider perspectives of human‐computer interaction for development research: opportunities and challenges. Paper presented at: 3rd African Human‐Computer Interaction Conference (AfriCHI 2021), Maputo, Mozambique; 2021. file:///C:/Users/Susan/AppData/Local/Temp/InsiderPerspectivesofHuman-ComputerInteractionforDevelopmentResearch-OpportunitiesandChallenges.pdf. Accessed May 13, 2021.

[27] Kozlakidis Z, Abduljawad J, Al Khathaami AM, Schaper L, Stelling J. Global health and data‐driven policies for emergency responses to infectious disease outbreaks. Lancet Global Health. 2020;8(11):e1361‐e1363. 10.1016/S2214-109X(20)30361-2. 32791118 PMC7417143

[28] Houreld K, Lewis D. In Africa, lack of coronavirus data raises fears of 'silent epidemic. *Reuters*; 2020. https://www.reuters.com/article/us-health-coronavirus-africa-data-insigh-idUSKBN24910L. Accessed May 13, 2021.

[29] Advisory Council on International Affairs (AIV) . *Digitalisation and youth employment in Africa*, Advisory Report to the Government of the Netherlands; 2020.

[30] Watson OJ, Abdelmagid N, Ahmed A, et al. *Characterising COVID‐19 epidemic dynamics and mortality underascertainment in Khartoum, Sudan*, report 39, Imperial College London; 2020, pp. 1–17. https://www.imperial.ac.uk/mrc-global-infectious-disease-analysis/covid-19/report-39-sudan/. Accessed May 13, 2020.

[31] Burke J. Lack of Covid data may leave African countries behind in vaccine rush. *The Guardian*. 2021. https://www.theguardian.com/world/2021/feb/15/lack-of-covid-data-may-leave-african-countries-behind-in-vaccine-rush. Accessed May 13, 2020.

[32] Mwananyanda L, Gill CJ, MacLeod W, et al. COVID‐19 deaths detected in a systematic post‐mortem surveillance study in Africa. MedRxiv. 2020. 10.1101/2020.12.22.20248327.

[33] van Elsland SL, Johns S. Majority of COVID‐19 deaths in Khartoum, Sudan are undetected. Imperial College London; 2020. https://www.imperial.ac.uk/news/209893/majority-covid-19-deaths-khartoum-sudan-undetected/. Accessed May 13, 2020.

[34] S. C. Inzaule, S. K. Tessema, Y. Kebede, A. E. Ogwell Ouma, and J. N. Nkengasong, “Genomic‐informed pathogen surveillance in Africa: opportunities and challenges,” Lancet Infect Dis, 2021, 1–19. https://www.thelancet.com/journals/laninf/article/PIIS1473‐3099(20)30939‐7/fulltext. Accessed May 13, 2021. 33587898 10.1016/S1473-3099(20)30939-7 PMC7906676

[35] Tessema SK, Inzaule SC, Christoffels A, et al. Accelerating genomics‐based surveillance for COVID‐19 response in Africa. Lancet Microb. 2020;1(6):e227‐e228. 10.1016/S2666-5247(20)30117-8 PMC7434434 32838350

[36] Ogunbiyi L. Data can become Nigeria's new ‘black gold,’ *Financial Times*; 2016. https://www.ft.com/content/67fcbca0-acf4-11e6-ba7d-76378e4fef24. Accessed May 13, 2021.

[37] United States Agency for Development (USAID) . Data [online], USAID; 2014. https://www.usaid.gov/global-health/health-systems-innovation/data. Accessed May 13, 2021.

[38] Ajadi S. *Digital health: a health system strengthening tool for developing countries*, Global System for Mobile Communications Association (GSMA). pp. 1–40; 2020. https://www.gsma.com/mobilefordevelopment/wp‐content/uploads/2020/11/Digital‐Health‐June‐2020.pdf. Accessed May 13, 2021.

[39] Broadband Commission for Sustainable Development . *Digital health: a call for government leadership and cooperation between ICT and health*, Working Group Health Report, roadband Commission for Sustainable Development; 2017. http://www.broadbandcommission.org/Documents/publications/WorkingGroupHealthReport-2017.pdf. Accessed May 13, 2021.

[40] Engels Z. Digital health apps in africa aim to revolutionize medical care [online], The Borgen Project Blog; 2020, https://borgenproject.org/digital-health-apps-in-africa/. Accessed May 13, 2021.

[41] Krah Eva FM, de Kruijf Johannes G. Exploring the ambivalent evidence base of mobile health (mHealth): A systematic literature review on the use of mobile phones for the improvement of community health in Africa. DIGITAL HEALTH. 2016;2:1–20. 10.1177/2055207616679264. PMC6001200 29942576

[42] Basajja M, van Reisen M, Oladipo F, Folorunsho S. FAIR Equivalency with Regulatory frameworks for Digital Health Policies in Uganda. Data Intell. 2021;3:in press.

[43] Basajja M, Mutwalibi N. Health information streams in clinics: the case of Uganda. Data Intell. 2021.

[44] Basajja, M. Information flows within hospitals in Uganda/African countries, Presentation at VODAN‐Africa Research Team Meeting, Leiden University; 2021

[45] van Reisen M. *International cooperation in the digital era*, Inaugural lecture on acceptance of position as professor of Computing for Society at the Universiteit Leiden; 2017. https://scholarlypublications.universiteitleiden.nl/access/item%3A2942216/view. Accessed May 13, 2021.

[46] Langen E. Diverging Worldviews, Diverging Worlds? Co‐Existence and Local Perspectives on Collaboration in the Pluralist Medical Setting of Macha. Zambia: Wageningen University; 2010.

[47] Chindoza K. Regulatory framework for e‐health data policies in zimbabwe: measuring FAIR equivalency. Data Intell. 2021;3.

[48] Abrahams L, Mbanaso U. State of internet security and policy in Africa. Paper presented at the 'First African Academic Network on Internet Policy', International Institute of Tropical Agriculture (IITA) Resort, Ibadan, Oyo, Nigeria; 2017. file:///C:/Users/Susan/AppData/Local/Temp/StateofInternetSecurityandPolicyinAfrica.pdf. Accessed May 13, 2021.

[49] Mudenda C, Johnson D, Parks L, van Stam G. Power instability in rural Zambia, case Macha BT—e‐Infrastructure and e‐Services for developing countries. In G. van Stam, T.F. Bissyande. Proceedings of International Conference, AFRICOMM 2013, Blantyre, Malawi, November 25–27, 2013, 2014, pp. 260–270. https://link.springer.com/book/10.1007%2F978-3-319-08368-1. Accessed May 13, 2021.

[50] Johnson D, van Stam G. The shortcomings of globalised internet technology in southern Africa, Paper presented at: Africomm 2016, Ouagadougou, Burkina Faso; 2016.

[51] Chawurura T, Manhibi R, Dijk J, van Stam G. Stocktaking digital health infrastructure in Zimbabwe, Paper Presented at: Public Health Conference (ICOPH 2020); 2020.

[52] Brijmohan Y, Manuhwa M, van Stam G. Regional trends in engineering: Africa. Engineering for Sustainable Development. Paris, France: UNESCO; 2021:172‐179.

[53] Regulation (EU) 2016/679. https://eur-lex.europa.eu/eli/reg/2016/679/oj. Accessed 16 May 2021.

[54] Pernot‐Leplay E. China's approach on data privacy law: a third way between the US and the EU? Penn State J Law Int Affairs. 2020;8(1):51–117.

[55] Al Tamimi & Company . Splinternet: do data localisation laws threaten the global Internet? [online], Lexology; 2017. https://www.lexology.com/library/detail.aspx?g=15527048-e5b8-43d1-ab6e-a934a4551a0a. Accessed May 13, 2021.

[56] Lanvin B. Conference Leveraging Digital Transformation in a post‐COVID Era: Panel focuses on Africa's digital opportunities and obstacles, striking an optimistic note [online], Portulans Institute Blog; 2020. https://portulansinstitute.org/leveraging‐digital‐transformation‐in‐a‐post‐covid‐era‐panel‐focuses‐on‐africas‐digital‐opportunities‐and‐obstacles‐striking‐an‐optimistic‐note/. Accessed May 13, 2021.

[57] Songwe DV. The role of digitalization in the decade of action for Africa [online], United Nations Conference on Tradae and Development (UNCTAD); 2020. https://unctad.org/news/role-digitalization-decade-action-africa. Accessed May 13, 2021.

[58] Reisen M, Stokmans M, Mawere M, et al. FAIR practices in Africa. Data Intell. 2020;2:246‐256. 10.1162/dint_a_00047.

[59] van Reisen M, Stokmans M, Basajja M, Onga'ayo O, Kirkpatrick C, Mons B. Towards the tipping point for FAIR implementation. Data Intell. 2020;2:264‐275. 10.1162/dint_a_00049.

[60] Stedman, C. What is data management and why is it important? [online]. TechTarget, October 2019. https://searchdatamanagement.techtarget.com/definition/data-management. Accessed May 13, 2021.

[61] East African Health Research Commission (EAHRC) . Digital REACH Initiative Roadmap, Digital Regional East African Community Health Initiative; 2018, p. 75. https://www.eahealth.org/sites/www.eahealth.org/files/content/attachments/2019‐02‐06/Digital‐REACH‐Initiative‐Roadmap_20171205_custom_size_0.pdf. Accessed May 14, 2021.

[62] Wilkinson MD, Dumontier M, Aalbersberg IJJ, et al. Comment: the FAIR guiding principles for scientific data management and stewardship. Sci Data. 2016;3:1‐9. 10.1038/sdata.2016.18. PMC4792175 26978244

[63] Carroll J, Bizer C, Hayes P, Stickler P. Named graphs. Web Seman Sci Services Agents World Wide Web. 2005;3:247‐267. 10.1016/j.websem.2005.09.001.

[64] National Institutes of Health . CEDAR [online], CEDAR. https://metadatacenter.org/. Accessed May 13, 2021.

[65] Berners‐Lee T. Weaving the Web: the Original Design and Ultimate Destiny of the World Wide Web. New York: HarperCollins; 2000.

[66] Fagnan K, Nashed Y, Perdue G, Ratner D, Shankar A, Yoo S. Data and Models: A Framework for Advancing AI in Science, Report of the Office of Science Roundtable on Data for AI, United States; 2019, doi: 10.2172/1579323

[67] Go FAIR . Go FAIR: IN‐Africa [online]. Go FAIR; 2020. https://www.go-fair.org/implementation-networks/overview/in-africa/. Accessed May 1, 2020.

[68] Go FAIR . GO FAIR Initiative [online]; 2019. https://www.go-fair.org/go-fair-initiative/. Accessed May 13, 2020.

[69] Nabudere H, The Digital REACH Initiative Roadmap . Towards a regional commitment to improve health outcomes through digital technology. Digital Health Symposium; 2018.

[70] S. Pettersen Nguyen, P. Nielsen, and J. Saebo, “The role of global standardization communities in shaping national health information architectures,” In J. Choudrie, M. Sirajul Islam, F. Wahid, J. M. Bass, J. Eka Priyatma (eds), Information and Communication Technologies for Development, Proceedings of 14th IFIP WG 9.4 International Conference on Social Implications of Computers in Developing Countries, ICT4D 2017 Yogyakarta, Indonesia, 2017, New York, NY: Springer; 2017.

[71] Jochems A, Deist TM, van Soest J, et al. Distributed learning: developing a predictive model based on data from multiple hospitals without data leaving the hospital: a real life proof of concept. Radiother Oncol J Eur Soc Ther Radiol Oncol. 2016;121(3):459‐467. 10.1016/j.radonc.2016.10.002. 28029405

[72] Purnama Jati PH. Improving Satu Data Indonesia with FAIR elements: a model to extend Satu Data Indonesia principles in COVID‐19 data management [masters thesis], Leiden University; 2020.

[73] Chindoza K, van Stam G, Mulingwa A, et al. E‐health implementation in Zimbabwe: an exploration to the usability of FAIR in data integration. Data Intell. 2021;3.

[74] Kawu AA, Joseph E, Abdullahi I, et al. FAIR principles and data regulatory frameworks for digital health in Nigeria. Data Intell. 2021;3.

[75] Taye GT, Amare SY, Gebreslassie GT, Medhanyie AA. FAIR Equivalency with Regulatory Frameworks for Digital health in Ethiopia. Data Intell. 2021;3.

[76] Thea EI, Nalugala R, Nandwa W, Obwanda F, Cartaxo AM. Regulatory framework for digital health data policies in Kenya: measuring FAIR equivalency. Data Intell. 2021;3.

[77] Mehl G, Tunçalp Ö, Ratanaprayul N, et al. WHO SMART guidelines: optimising country‐level use of guideline recommendations in the digital age. Lancet Digital Health. 2021;7500(21):8‐11. 10.1016/S2589-7500(21)00038-8. 33610488

[78] Lin Y, Purnama Jati PH, Aktau A, Nodehi S, van Reisen M. Implementation of FAIR principles in selected non‐English speaking geographies. Data Intell. 2021;3.

[79] Basajja M, Suchanek M, Taye GT. et al. Proof of concept and horizons on deployment of FAIR in the COVID‐19 pandemic. Data Intell. 2021;3.

[80] M. van Reisen and F. Oladipo, “Proof of Concept Developed by VODAN Africa and Asia [online],” Leiden, The Netherlands: Go‐FAIR Foundation, 2020. https://www.go-fair.org/2020/10/27/proof-of-concept-developed-by-vodan-africa-and-asia/. Accessed May 13, 2020.

[81] Amare SY, Taye GT, Van Stam G. Freedom to operate and convergence of tools. Data Intell. 2021;3.

[82] Tayelle GT, Amare SY. Beyond eCRF: requirements for implementation in regular health systems. Data Intell. 2021;3.

[83] van Reisen M, Oladipo F, Osigwe O. VODAN Africa timeline planning: march, Presentation (unpublished); March 2021.

[84] Ghardallou M, Wirtz M, Folorunso S, et al. Expansion to publication of non‐patient COVID‐data: Toward the FAIRification of Migrants’ Data in Tunisia. Data Intell. 2021;3.

[85] Folorunso S, Ogundepo EA, Basajja M, et al. Nigeria COVID‐19 FAIR data analytics with machine learning models. Data Intell. 2021;3.

[86] Holst C, Sukums F, Radovanovic D, Ngowi B, Noll J, Winkler AS. Sub‐Saharan Africa – the new breeding ground for global digital health. Lancet Digital Health. 2020;2(4):e160‐e162. 10.1016/S2589-7500(20)30027-3. 33328076

[87] Akindele AT, Tayo AO, Taye GT, et al. The Impact of COVID‐19 on distance education and the new potentials of distance education. Data Intell. 2021;3.

[88] Oladipo F, Folorunso S, Ogundepo EA, Osigwe EO, Akindele AT. Curricula development for FAIR data stewardship. Data Intell. 2021;3.

[89] Kampala International University . DISH: Digital innovation and skills hub; 2021. https://codeesa.kiu.ac.ug/dish.php. Accessed May 13, 2020.

[90] Kampala International University . Data science through GO FAIR in Africa: a new generation internet of data & services; 2020. https://www.kiu.ac.ug/engagements.php?i=data-science-through-go-fair-in-africa-a-new-generation-internet-of-data-and-services. Accessed May 1, 2020.

[91] Coyne R. Wicked problems revisited. Design Stud. 2005;26:5‐17. 10.1016/j.destud.2004.06.005.

[92] Elixir . ELIXIR deposition databases for biomolecular data [online], Elixir. https://elixir‐europe.org/platforms/data/elixir‐deposition‐databases. Accessed May 13, 2021.

