# Supplementary material for: Design of a FAIR digital data health infrastructure in Africa for COVID‐19 reporting and research
Source: Adv Genet (Hoboken). 2021 Jun 11;2(2):e10050. doi: 10.1002/ggn2.10050 (PMC8420285; doi:10.1002/ggn2.10050)
Supplement: Supplementary file 1 — Supplementary TPR File [file GGN2-2-e10050-s001.pdf]

ADVANCED GENETICS
Open Access

Design of a FAIR Digital Data Health Infrastructure in Africa for COVID-19 Reporting and Research

Mirjam van Reisen*, Francisca Oladipo, Mia Stokmans, Mouhamed Mpezamihgo, Sakinat Folorunso, Erik Schultes, Mariam Basajja, Aliya Aktau, Samson Yohannes Amare, Getu Tadele Taye, Putu Hadi Purnama Yati, Kudakwashe Chindoza, Morgane Wirtz, Meriem Ghardallou, Gertjan van Stam, Wondimu Ayele, Reginald Nalagala, Ibrahim Abdullahi, Obinna Osigwe, John Graybeal, Araya Abrha Medhanyie, Abdullahi Abubakar Kawu, Fenghong Liu, Katy Wolstencroft, Erik Flikkenschild, Yi Lin, Joëlle Stocker, Mark A. Musen

*Corresponding

Review timeline:	
Date Submitted:	30-Mar-2021
Editorial Decision:	10-May-2021 Minor Revision
Revision Received:	20-May-2021
Accepted:	21-May-2021

Editor: Myles Axton

Initial Editorial Evaluation	31-Mar-2021

Summary

The VODAN architecture provides a first practical implementation of WHO SMART Guidelines and identifies the possibility of interoperability between clinical data and research data, while data remain in residence where generated.

Here is a report of the pilot project to build secure, FAIR data network for clinical reporting and viral and genomic research in Africa. Here is broad agreement on how to implement - in resource poor environments - principles of data perdurance and personal consented control by the patient or research subject while permitting transnational epidemiological research to improve decision making and informatics infrastructure. Two elements were key, the East Africa Open Science Cloud for Health and adoption of FAIR data by the VODAN covid-19 reporting consortium. Obstacles overcome and recommendations made from this study are the use of CEDAR templates to augment the metadata creation at the participating centers as well as for reuse by other partners. WHO paper templates proved difficult to replace with a FAIR data implementation and impossible to query without CEDAR's support.

Scope

Do the research, methods or topics fit within the aims of this, or another journal?

Yes, this is key to collaborative genomic research as well as medical informatics interoperability. Need for genome-based virus surveillance [83, 84]

Is this best published as a Research Perspective or Article (reviewers to comment)?

This is a literature and database-based Perspective making recommendations for best practice. Some of the related papers in press at *Data Intelligence* would probably be of interest to the reviewers of this work if they can be made available in preprint or attached to the uploaded manuscript in this database.
This is a model for equitable access to and reuse of research genomic data and disease phenotypes from patients and research subjects.

1st Peer Review	01-Apr-2021 to 10-May-2021

Reviewer #1

General comments:

The work is a significant and novel contribution to the field. It is well organized and comprehensively described. The research addresses the urgent problem of ownership and management of data in non-Western contexts from an empirical point of view. It does it from the perspective of the African continent. I want to mention that the extensive list of authors from different affiliations and locations adds to the paper's richness and self-represent the principle of inclusion. The manuscript is scientifically sound.

Minor improvements:

1.1 Since the manuscript is based on project results, a paragraph or two to situate the project initiative and power for action (funders, management, original aims) will enrich the contextualization somewhere between sections 3 and 5.

1.2 Although it presents appropriate references, they need to be revised into the correct referencing style, as most of the references are incomplete (i.e., page number, DOI, etc.). There are self-citations, but they are not inappropriate and show the authors' existing knowledge of the topic.

Reviewer #2

The authors report on the progress of designing an inclusive health data management platform for Africa. They describe the complex landscape within which the design process was undertaken, including both social and technical challenges, and the broadly consultative approach to achieving agreement on, at least, objectives and approaches to achieve them. This is all done with an overarching goal of being, to the greatest extent possible, compliant with the FAIR Principles for data publication, to ensure that the final product achieves larger goals of maximizing the utility of the data, in particular, for the African participants themselves. The paper is well-thought-out and written to a high standard of scholarship and language quality.

2.1 I have no expertise in ethnography - my knowledge is limited to only what I have learned in rare but enjoyable personal conversations with the lead author. As such, I will focus my comments primarily on the structure of the paper, their interpretation of FAIR, and on some technical decisions that I had some concerns with.

2.2 In general, I found the paper to be a bit longer than it needed to be, and this was often due to repetition. A particularly notable example is in section 9, where the same list is reiterated twice on the same page. However, repetition is common throughout the manuscript, with repeated references to, for example, "wicked problems".

2.3 With respect to the interpretation of FAIR (especially section 4), there are several distinct ideas being conflated that really should be more clearly separated. FAIR does not "build on the semantic web through linked data" - in fact, FAIR does not propose a technology at all. Many (not all) people who attempt to implement FAIR do so using Linked Data. When that Linked data is grounded in Ontologies, it can become a part of the Semantic Web; however, this is a consequence of the selection of a technology to implement FAIR, more than it is related to FAIR itself.

2.4 The following sentence is hard to interpret, and (if my interpretation is correct) is also not correct: "The guidelines promote data localization through the repository of data within residence; data visiting by exposure of the data through reachability over the Internet; machine readability by making use of linked-data approaches under the proviso that data is readable by both humans and machines; and data convergence by introducing controlled vocabularies within communities that collaborate on data." FAIR says nothing about where data should be stored. The idea of "data visiting" is a behaviour that is enabled by FAIR, but is not part of FAIR itself - I suspect that the reason that the ideas are being conflated here is that the FAIR for Africa initiative has been working with the Personal Health Train initiative, the latter of which, has defined a mechanism for "data visiting" - however, this is an idea that is coming from that project, not from FAIR.

2.5 "through controlled vocabularies of data [55] held in residence" - this sentence also appears in section 4, and sounds very odd until the implication of "held in residence" becomes clearer later in the manuscript. In fact, it is in many ways antithetical to the objectives of FAIR to have a controlled vocabulary "held in residence", since FAIR vocabularies are intended to be as widely used as possible. Having read the manuscript a few times, I think I understand what the author is saying - that some vocabularies had to be built specifically for FAIR for Africa, and perhaps at an even more fine-grained level to deal with the

ethnographic aspects of the project. I would therefore suggest that this odd phrase be removed from the sentence at this point in the manuscript, and then raised later when it can be explained properly.

2.6 "The FAIR Principles provide a spectrum of FAIR Data, which can be referred to as degrees of FAIRness." perhaps re-word this as "The modular FAIR Principles provide FOR a spectrum of levels of compliance, which can be..."

2.7 "The FAIR Principles draw on the work of Stanford University work with CEDAR Workbench for Open Science" - this is not really true. Investigators from the CEDAR project were certainly in the room during the Lorenz workshop, but it is a stretch to suggest that the Principles "draw on" that work. In fact, the opposite would be more accurate.

2.8 "CEDAR Workbench for Open Science and Bioportal, among others, which is identified as fully FAIR-compliant [56]." Again, this isn't true. They "self-identify" as being fully FAIR-compliant, but that is a statement on a web page that has not been challenged by peer-review as far as I am aware. In fact, using existing FAIR evaluation software, I am not aware of any resource that has passed all tests, and therefore there may be no resource on earth at this time that is "fully FAIR compliant". Combining that with the fact that there is no objective standard for measuring FAIRness, and this statement becomes unsupportable and is more speculation/marketing than a scholarly observation.

2.9 Staying on the topic of CEDAR: The manuscript describes the selection of CEDAR (over the DSWizard) as a metadata capture interface. I happen to agree with the authors that CEDAR's interface is superior to DSW, and much easier to configure; however, I think the authors do not sufficiently discuss the shortcomings of CEDAR, particularly in the context of this manuscript, in which data "ownership" is such an important issue! To my knowledge (after exploring this over the past few days to ensure that my knowledge is up-to-date) there is no deployment of the CEDAR software outside of Stanford University. There is, for example, no Docker image, that would allow the system to be deployed within a hospital's protected data environment, and no installation instructions on the CEDAR GitHub. Moreover, both the templates and the metadata they capture, are stored on Stanford's servers in the United States (as far as I am aware...?). Given that data ownership, privacy, and control are such important features of this manuscript, it would seem that CEDAR might be the worse of the two choices (given that DSW can be deployed locally, and all data is stored locally). I would appreciate the author giving a more balanced discussion of this choice, as it concerned me somewhat when I read it. I would also suggest not referring to the CEDAR website as a scholarly reference, since those statements have (often) not been vetted.

2.10 "The data could not be queried, despite the availability of a FAIR Data Point". This seems unlikely to be accurate. Data in a FAIR Data Point is, almost by definition, queryable. Are you saying that the lack of standardization made it difficult to construct queries consistently from one site to another?

2.11 General structural comment: The idea of "turning points" arises repeatedly in the manuscript, and I felt that these were very interesting statements/observations. I would welcome those to be more highlighted - perhaps in a figure, or even better, perhaps as a guiding structure for the manuscript itself (though I understand that this would be a significant re-write! Nevertheless, these moments in the project seem to be more significant than the attention they are given in the text.)

2.12 A final small note about "jargon". While I appreciate that jargon is frequently used to increase the efficiency of communication within a specialist community, I feel it is inappropriate to use jargon in a manuscript submitted to a journal like Advanced Genetics, which will have a readership quite distant from the expert community that this paper represents. Phrases like "...and are coping with different health orientations that co-exist in communities" are not easily interpreted by researchers outside of your specialist domain. It would be useful to give this manuscript to a non-domain-expert and simply have them highlight sentences that seem to assume highly specialist knowledge.

Overall, I congratulate the authors on this work. It was an enjoyable read, and especially interesting to see how it has evolved since my early days as a "fly on the wall" listening to the conversations that gave birth to this great initiative!

[In accordance with journal policy, this signed review can be seen in Publons once the review process is complete - ED

1st Editorial Decision	10-May-2021

Editorial decision: The editor recommends to resubmit with minor revisions with recommendations below and in the marked-up manuscript.

Editor's understanding of the reviews
Reviewer #1 recommends Minor Revision
Reviewer #2 recommends Minor Revision

**Reviewer comments**	**Editor recommendation**	**Author reply**	**Changes to Manuscript**
2.3 there are several distinct ideas being conflated<br>2.4 the Personal Health Train initiative... has defined a mechanism for "data visiting"<br>2.5 through controlled vocabularies of data held in residence<br>2.6 degrees of FAIRness<br>2.10 Data in a FAIR Data Point is, almost by definition, queryable	ED1 please distinguish the aspirational FAIR principles from implementation technologies, related initiatives, local vocabularies, FAIR compliance vs levels of FAIR, local difficulties vs definitions of operation		
1.2 correct referencing style<br>2.7 CEDAR Workbench for Open Science<br>2.8 there may be no resource on earth at this time that is "fully FAIR compliant<br>2.9 selection of CEDAR (over the DSWizard) as a metadata capture interface	ED2 CEDAR URL can certainly be cited, but no claims of its FAIR compliance can currently be made. The selection of one metadata capture interface over another can be mentioned with report of advantages and disadvantages		
2.11 The idea of "turning points" arises repeatedly in the manuscript.. perhaps as a guiding structure for the manuscript itself<br><br>1.1 a paragraph or two to situate the project initiative and power for action (funders, management, original aims)	ED3 We welcome an additional Table or Box if that is expeditious to assemble, documenting decision points where the project was re-evaluated, with the reason for the decision and its consequences, new implementation or learning.<br><br>The resulting Action Points for original aims, management and funders, as well as for the intended research and clinical and epidemiological end users can also be in this Table as well as in the recommended paragraphs.		
2.12 highlight sentences that seem to assume highly specialist knowledge	ED4 Emphasize the need for this FAIR network for genomic epidemiology in this setting. Remove computational, ontological or ethnological jargon that is not useful for genetics and genomics or for universal metadata collaboration according to basic FAIR principles.<br>The internal projects and workings of GO-FAIR can be omitted or just cited.		
2.2 repetition is common throughout the manuscript	ED5 See marked up manuscript for three suggested Boxes. Repetition of the points made in the boxes and other repetition can be removed		

Author's Response to 1st Review	20-May-2021

Reviewer comments	Editor recommendation	Author reply	Changes to Manuscript
2.3 there are several distinct ideas being conflated<br>2.4 the Personal Health Train initiative... has defined a mechanism for "data visiting"<br>2.5 through controlled vocabularies of data held in residence<br>2.6 degrees of FAIRness<br>2.10 Data in a FAIR Data Point is, almost by definition, queryable	ED1 please distinguish the aspirational FAIR principles from implementation technologies, related initiatives, local vocabularies, FAIR compliance vs levels of FAIR, local difficulties vs definitions of operation	- The criticism is correct. I have separated FAIR, PHT and VODAN-Africa.<br>- I have separated the idea of data visiting<br>- I have separated the idea of data in repository where data is produced<br>- I have reformulated degrees of FAIRness<br>- I do not agree with 2.10. In our research we speak of interoperability of the data-instances itself, not information about the data. I hope this is clarified.	
1.2 correct referencing style<br>2.7 CEDAR Workbench for Open Science<br>2.8 there may be no resource on earth at this time that is "fully FAIR compliant<br>2.9 selection of CEDAR (over the DSWizard) as a metadata capture interface	ED2 CEDAR URL can certainly be cited, but no claims of its FAIR compliance can currently be made. The selection of one metadata capture interface over another can be mentioned with report of advantages and disadvantages	• I have edited the references using the style in MLA<br>• I have deleted the word fully so that it just reads 'compliant' now.<br>• Added 'self-identified' to CEDAR as FAIR compliant (since it is a spectrum, there is not actually a way to measuring full compliance)<br>• Selection of CEDAR over DSWizard has been explained more thoroughly	
2.11 The idea of "turning points" arises repeatedly in the manuscript.. perhaps as a guiding structure for the manuscript itself<br><br>1.1 a paragraph or two to situate the project initiative and power for action (funders, management, original aims)	ED3 We welcome an additional Table or Box if that is expeditious to assemble, documenting decision points where the project was re-evaluated, with the reason for the decision and its consequences, new implementation or learning.<br><br>The resulting Action Points for original aims, management and funders, as well as for the intended research and clinical and epidemiological end users can also be in this Table as well as in the recommended paragraphs.	- Provided a more precise definition of the concept 'turning points"<br>- Added overview table<br>- Added all turning points in Box 3<br>- I have added all funders to the list of acknowledgements	
2.12 highlight sentences that seem to assume highly specialist knowledge	ED4 Emphasize the need for this FAIR network for genomic epidemiology in this setting. Remove computational, ontological or ethnological jargon that is not useful for genetics and genomics or for universal metadata collaboration according to basic FAIR principles.<br>The internal projects and workings of GO-FAIR can be omitted or just cited.	- The article has been edited to avoid jargon and simplify the ideas expressed.<br>- The order of the sections is changed with the section on "pre-development" moved up as new section 2.	

2.2 repetition is common throughout the manuscript	ED5 See marked up manuscript for three suggested Boxes. Repetition of the points made in the boxes and other repetition can be removed	- Removal of repetition across the text<br>- Shortening of text- creating greater cogency of the principal thoughts<br>- Inclusion of key-visuals and boxes	

Reviewer #1

General comments:

The work is a significant and novel contribution to the field. It is well organized and comprehensively described. The research addresses the urgent problem of ownership and management of data in non-Western contexts from an empirical point of view. It does it from the perspective of the African continent. I want to mention that the extensive list of authors from different affiliations and locations adds to the paper's richness and self-represent the principle of inclusion. The manuscript is scientifically sound.

Minor improvements:

1.1 Since the manuscript is based on project results, a paragraph or two to situate the project initiative and power for action (funders, management, original aims) will enrich the contextualization somewhere between sections 3 and 5.

1.2 Although it presents appropriate references, they need to be revised into the correct referencing style, as most of the references are incomplete (i.e., page number, DOI, etc.). There are self-citations, but they are not inappropriate and show the authors' existing knowledge of the topic.

Reviewer #2

The authors report on the progress of designing an inclusive health data management platform for Africa. They describe the complex landscape within which the design process was undertaken, including both social and technical challenges, and the broadly consultative approach to achieving agreement on, at least, objectives and approaches to achieve them. This is all done with an overarching goal of being, to the greatest extent possible, compliant with the FAIR Principles for data publication, to ensure that the final product achieves larger goals of maximizing the utility of the data, in particular, for the African participants themselves. The paper is well-thought-out and written to a high standard of scholarship and language quality.

2.1 I have no expertise in ethnography - my knowledge is limited to only what I have learned in rare but enjoyable personal conversations with the lead author. As such, I will focus my comments primarily on the structure of the paper, their interpretation of FAIR, and on some technical decisions that I had some concerns with.

2.2 In general, I found the paper to be a bit longer than it needed to be, and this was often due to repetition. A particularly notable example is in section 9, where the same list is reiterated twice on the same page. However, repetition is common throughout the manuscript, with repeated references to, for example, "wicked problems".

2.3 With respect to the interpretation of FAIR (especially section 4), there are several distinct ideas being conflated that really should be more clearly separated. FAIR does not "build on the semantic web through linked data" - in fact, FAIR does not propose a technology at all. Many (not all) people who attempt to implement FAIR do so using Linked Data. When that Linked data is grounded in Ontologies, it can become a part of the Semantic Web; however, this is a consequence of the selection of a technology to implement FAIR, more than it is related to FAIR itself.

2.4 The following sentence is hard to interpret, and (if my interpretation is correct) is also not correct: "The guidelines promote data localization through the repository of data within residence; data visiting by exposure of the data through reachability over the Internet; machine readability by making use of linked-data approaches under the proviso that data is readable by both humans and machines; and data convergence by introducing controlled vocabularies within communities that collaborate on data." FAIR says nothing about where data should be stored. The idea of "data visiting" is a behaviour that is enabled by FAIR, but is not part of FAIR itself - I suspect that the reason that the ideas are being conflated here is that the FAIR for Africa initiative has been working with the Personal Health Train initiative, the latter of which, has defined a mechanism for "data visiting" - however, this is an idea that is coming from that project, not from FAIR.

2.5 "through controlled vocabularies of data [55] held in residence" - this sentence also appears in section 4, and sounds very odd until the implication of "held in residence" becomes clearer later in the manuscript. In fact, it is in many ways antithetical to the objectives of FAIR to have a controlled vocabulary "held in residence", since FAIR vocabularies are intended to be as widely used as possible. Having read the manuscript a few times, I think I understand what the author is saying - that some vocabularies had to be built specifically for FAIR for Africa, and perhaps at an even more fine-grained level to deal with the ethnographic aspects of the project. I would therefore suggest that this odd phrase be removed from the sentence at this point in the manuscript, and then raised later when it can be explained properly.

2.6 "The FAIR Principles provide a spectrum of FAIR Data, which can be referred to as degrees of FAIRness." perhaps re-word this as "The modular FAIR Principles provide FOR a spectrum of levels of compliance, which can be..."

2.7 "The FAIR Principles draw on the work of Stanford University work with CEDAR Workbench for Open Science" - this is not really true. Investigators from the CEDAR project were certainly in the room during the Lorenz workshop, but it is a stretch to suggest that the Principles "draw on" that work. In fact, the opposite would be more accurate.

2.8 "CEDAR Workbench for Open Science and Bioportal, among others, which is identified as fully FAIR-compliant [56]." Again, this isn't true. They "self-identify" as being fully FAIR-compliant, but that is a statement on a web page that has not been challenged by peer-review as far as I am aware. In fact, using existing FAIR evaluation software, I am not aware of any resource that has passed all tests, and therefore there may be no resource on earth at this time that is "fully FAIR compliant". Combining that with the fact that there is no objective standard for measuring FAIRness, and this statement becomes unsupportable and is more speculation/marketing than a scholarly observation.

I have deleted the word ‘fully’.

2.9 Staying on the topic of CEDAR: The manuscript describes the selection of CEDAR (over the DSWizard) as a metadata capture interface. I happen to agree with the authors that CEDAR's interface is superior to DSW, and much easier to configure; however, I think the authors do not sufficiently discuss the shortcomings of CEDAR, particularly in the context of this manuscript, in which data "ownership" is such an important issue! To my knowledge (after exploring this over the past few days to ensure that my knowledge is up-to-date) there is no deployment of the CEDAR software outside of Stanford University. There is, for example, no Docker image, that would allow the system to be deployed within a hospital's protected data environment, and no installation instructions on the CEDAR GitHub. Moreover, both the templates and the metadata they capture, are stored on Stanford's servers in the United States (as far as I am aware...?). Given that data ownership, privacy, and control are such important features of this manuscript, it would seem that CEDAR might be the worse of the two choices (given that DSW can be deployed locally, and all data is stored locally). I would appreciate the author giving a more balanced discussion of this choice, as it concerned me somewhat when I read it. I would also suggest not referring to the CEDAR website as a scholarly reference, since those statements have (often) not been vetted.

2.10 "The data could not be queried, despite the availability of a FAIR Data Point". This seems unlikely to be accurate. Data in a FAIR Data Point is, almost by definition, queryable. Are you saying that the lack of standardization made it difficult to construct queries consistently from one site to another?

2.11 General structural comment: The idea of "turning points" arises repeatedly in the manuscript, and I felt that these were very interesting statements/observations. I would welcome those to be more highlighted - perhaps in a figure, or even better, perhaps as a guiding structure for the manuscript itself (though I understand that this would be a significant re-write! Nevertheless, these moments in the project seem to be more significant than the attention they are given in the text.)

2.12 A final small note about "jargon". While I appreciate that jargon is frequently used to increase the efficiency of communication within a specialist community, I feel it is inappropriate to use jargon in a manuscript submitted to a journal like Advanced Genetics, which will have a readership quite distant from the expert community that this paper represents. Phrases like "...and are coping with different health orientations that co-exist in communities" are not easily interpreted by researchers outside of your specialist domain. It would be useful to give this manuscript to a non-domain-expert and simply have them highlight sentences that seem to assume highly specialist knowledge.

Overall, I congratulate the authors on this work. It was an enjoyable read, and especially interesting to see how it has evolved since my early days as a "fly on the wall" listening to the conversations that gave birth to this great initiative!

[In accordance with journal policy, this signed review can be seen in Publons once the review process is complete - ED]

2nd Editorial Decision	21-May2021

The manuscript has now been revised in accordance with reviewer and editor recommendations and we have now decided to accept the revised manuscript.
